# A Review on Biphasic Calcium Phosphate Materials Derived from Fish Discards

**DOI:** 10.3390/nano11112856

**Published:** 2021-10-26

**Authors:** Liviu Duta, Gabriela Dorcioman, Valentina Grumezescu

**Affiliations:** Lasers Department, National Institute for Lasers, Plasma and Radiation Physics, 077125 Magurele, Romania; liviu.duta@inflpr.ro (L.D.); gabriela.dorcioman@inflpr.ro (G.D.)

**Keywords:** biphasic calcium phosphates, fish discards, bone-tissue engineering

## Abstract

This review summarizes the results reported on the production of biphasic calcium phosphate (BCP) materials derived from fish wastes (i.e., heads, bones, skins, and viscera), known as fish discards, and offers an in-depth discussion on their promising potential for various applications in many fields, especially the biomedical one. Thus, considerable scientific and technological efforts were recently focused on the capability of these sustainable materials to be transformed into economically attractive and highly valuable by-products. As a consequence of using these wastes, plenty of beneficial social effects, with both economic and environmental impact, will arise. In the biomedical field, there is a strong and continuous interest for the development of innovative solutions for healthcare improvement using alternative materials of biogenic origin. Thus, the orthopedic field has witnessed a significant development due to an increased demand for a large variety of implants, grafts, and/or scaffolds. This is mainly due to the increase of life expectancy and higher frequency of bone-associated injuries and diseases. As a consequence, the domain of bone-tissue engineering has expanded to be able to address a plethora of bone-related traumas and to deliver a viable and efficient substitute to allografts or autografts by combining bioactive materials and cells for bone-tissue ingrowth. Among biomaterials, calcium phosphate (CaP)-based bio-ceramics are widely used in medicine, in particular in orthopedics and dentistry, due to their excellent bioactive, osteoconductive, and osteointegrative characteristics. Recently, BCP materials (synthetic or natural), a class of CaP, which consist of a mixture of two phases, hydroxyapatite (HA) and beta tricalcium phosphate (β-TCP), in different concentrations, gained increased attention due to their superior overall performances as compared to single-phase formulations. Moreover, the exploitation of BCP materials from by-products of fish industry was reported to be a safe, cheap, and simple procedure. In the dedicated literature, there are many reviews on synthetic HA, β-TCP, or BCP materials, but to the best of our knowledge, this is the first collection of results on the effects of processing conditions on the morphological, compositional, structural, mechanical, and biological properties of the fish discard-derived BCPs along with the tailoring of their features for various applications.

## 1. Introduction

The damage or even the loss of an organ because of aging or trauma has lately become an important issue that needs rapid solutions. In this respect, two possible ways to solve these problems are either to repair or to replace the damaged tissue. Keeping in mind the latest technological advances in the field of biomaterials used for bone-tissue engineering, the latter have to meet three important functions: (i) to be porous, to have a hydrophilic behavior, and be able to hold water and essential body fluids; (ii) to be able to evenly transfer the mechanical load, with reduced friction and wear; and (iii) to support protein adhesion, cell proliferation, and differentiation.

Over the last decades, the biomedical field has witnessed a significant development due to an increased demand for a large variety of bone implants, grafts, and/or scaffolds. This is mainly because of the increase of life expectancy and higher frequency of injuries and diseases. As a consequence, the domain of bone-tissue engineering has expanded to be able to address a plethora of related traumas and to deliver a viable and efficient substitute to allografts or autografts by combining bioactive materials and cells for bone-tissue ingrowth. The surface functionalization of implants with bioactive materials is currently of high interest to the medical field. The bioactive materials represent a vast bioengineering research field with tremendous interest for the production of durable implants and bone substitutes able to bypass integration difficulties. Besides optimal surgical handiness and mechanical properties, implantable biomaterials for bone regeneration should be highly biocompatible and favorable to cellular adhesion, proliferation, and differentiation to sustain rapid tissue healing after implantation. The global implantable devices market was valued at $72,265 million in 2015 and is foreseen to reach $116,300 million by 2022 [1].

Among synthetic biomaterials, calcium phosphate (CaP)-based bio-ceramics, such as hydroxyapatite (HA), tricalcium phosphate (TCP), and a mixture of these two, called biphasic calcium phosphate (BCP), are widely investigated in medicine, in particular in orthopedics and dentistry [2,3]. Due to their proven excellent bioactivity, osteoconductivity, and osteointegration behaviors, one of the most important applications of these bio-ceramics is to coat various metallic implants [4], with the aim to promote bone growth on their surfaces [5]. The most frequently used CaP is HA, Ca_10_(PO_4_)_6_(OH)_2_, due to its ability to form strong bonds with the host bone tissues [6].

There are two main categories of methods used to obtain HA: (i) the first one involves the use of inorganic synthesis by different chemical routes (i.e., hydrothermal [7], co-precipitation [8], or sol-gel [9]). It should be emphasized that some of these methods could imply the use of complex processes, which might generate hazardous wastes. In addition, these approaches are time-consuming and could involve some rather high preparation costs [10,11]. Moreover, the chemistry of synthetic HA does not entirely reproduce the composition of the bone mineral phase [12]. (ii) As a consequence, researchers found a simple, reliable and highly productive alternative to classical chemical routes, i.e., to extract it from sustainable CaP resources. One should note that the most important primary natural reservoirs of CaP are either bones (of mammalian or fish origin [13,14,15]) or biogenic sources (egg-shells [16], whelks, or sea-shells [17]), often treated as food industry wastes only [18]. Fish bones are rich in calcium, carbonate, and phosphate and could be reliable sources for the production of natural HA (BioHA). Moreover, as compared to HA derived from bovine bones, the one obtained from fish sources is thermally stable at temperatures ~1200 °C [2]. Besides the low production cost, one great advantage of this fabrication route is the preservation of the source material’s compositional and structural properties [19]. Furthermore, the as-fabricated BioHA materials are well-suited to achieve a good synergy with the biological media since they already contain traces of beneficial ions [2,4], which play a key role in the proper adjustment of their properties (i.e., solubility, surface chemistry, morphology, biocompatibility) to have a stimulatory effect on bone formation both in vitro and in vivo [3,20,21,22]. The mineral constituent of the vertebrate skeletal system mainly consists of a carbonated, non-stoichiometric Ca-deficient material with a reduced degree of crystallinity. In the literature, it was reported that CaPs obtained from biogenic resources, with a nonstoichiometric composition and a disordered nanostructure [23], are more biocompatible [24] and demonstrate a better metabolic activity in comparison to stoichiometric ones [25]. Thus, BioHA differs from synthetic HA in terms of composition, stoichiometry, degree of crystallinity, degradation rate, and overall biological performance, being therefore from the biomimetic point of view more appropriate to repair the skeletal system. It was reported that HA obtained from low production cost resources, such as fish discards, animal bones, and egg-shells, can lead to overall physical properties and biological response comparable or even improved in comparison to synthetic ones. This is mainly due to their resemblance with bone apatite [26,27] and a considerable hetero-ionic exchange [28]. Besides the huge cut of production costs, the fabrication of bioapatite from natural resources secures the preservation of the source material’s composition and structure [19] and allows for a superior biomimicry. The production of quite cheap medical devices becomes accessible on these bases [16,29,30,31].

HA has a poor biodegradation rate, which determines its combination with other more bio-degradable bone phases at an appropriate ratio. On the other hand, β-TCP is characterized by high chemical stability, good biological activity, and favorable biodegradation rate. Despite these advantages, its degradation rate is difficult to control, with an unwanted effect on the formation rate of the newly formed bone [32].

The first mention in literature about tricalcium phosphate dates to 1975, belonging to Nery and his collaborators [33]. They reported on clinical application as bone substitutes in surgically created “infrabony” periodontal defects in animals. They found by X-ray diffraction (XRD) that the material was a mixture of 20% HA and 80% β-TCP [34].

The term BCP was first introduced by Moore et al. [35] and Ellinger et al. [36], who reported its application in periodontal osseous defects. Based on these pioneering studies, LeGeros [37] and Daculsi et al. [38] opened the pathway towards the preparation and clinical application of BCP. BCPs represent a class of bone-substitute materials, which generally consist of a mixture of two CaP phases in different concentrations: a soluble phase (i.e., β-TCP), which possesses a favorable degradation rate and a higher bioactivity, and a stable one (HA), which ensures stability, lower absorption rate, and excellent mechanical properties [39]. BCP mixtures are of two major types: (i) CaP phases with similar molar Ca/P ratio (e.g., α-TCP and β-TCP, Ca/P = 1.5 for both) and (ii) CaP phases with different molar ratio (e.g., β-TCP and HA, Ca/P = 1.5 and 1.67, respectively).

In the case of coatings, Behera et al. [40] postulated that the weight percentage (wt.%) of individual phases present in a surface can be calculated using the following formula:

(1) $$W_{\mathrm{HA}}=\left\{\frac{1}{\left[1+1.265(I_{\beta \text{-}\mathrm{TCP}}/I_{\mathrm{HA}})\right]}\right\}\times 100$$

where *W*_HA_, *I*_β-TCP_, and *I*_HA_ denote the wt.% of HA, intensity of strongest reflection of β-TCP, and HA, respectively.

Therefore, due to their superior characteristics over single-phase formulations (e.g., pure HA, β-TCP, or other CaP bioceramics), in terms of osteoconduction potential without any growth factors [41,42], BCP materials are regarded with much interest [43]. This BCP is a very suitable material for bone-substitute applications because while the HA provides a scaffold for new bone formation via osteoconduction, the reabsorption of β-TCP oversaturates the local environment with calcium and phosphate ions, which accelerates the new bone formation [44]. An adequate balance between HA and β-TCP can determine the mechanical properties, biodegradability, and overall stability of the final phases [45,46]. Furthermore, BCP exhibits higher hydrophobicity than HA but lower than β-TCP [47]. Thus, the adsorption of protein is enhanced in the case of BCP due to hydrophobic interactions, which results in an improved osteopromotion as compared to HA [47]. This control of the properties warrants a good stability while promoting an effective bone ingrowth [39,48,49]. Thus, by proper tailoring the phase concentrations, one can fabricate BCP materials able to preserve their configuration over prolonged exploitation times [50] for the restoration of various bone defects in high-load-bearing areas. Moreover, it was demonstrated that, by incorporation of other materials (such as bioglasses, BG) into CaPs, the mechanical strength could be enhanced. In addition, the bioactivity of CaP is lower when compared to that of BG. Thereby, a combination of this type of materials creates bone grafts with improved biological responses [51].

One should warn here that the available mineral resources are nowadays threatened and strained by the rapid demographic increase and economic growth. The access to unconventional, sustainable resources became therefore opened and encouraged for future economic development and prosperity [52]. Fish and shellfish are generally used for consumption, and it was estimated that almost half of more than 90 million tons of these marine fishes caught globally each year are discarded by the fish processing industry (FPI) [13,53,54]. This is not only harmful for fish species themselves but can also negatively contribute to the environmental pollution by altering the marine environment and even favoring the development of parasites, such as anisakis [55]. Fish wastes are highly perishable, and therefore, for their complete utilization, one should (i) develop systems able to sort and handle the rest raw materials directly onboard; (ii) identify or even discover safe and cost-effective preservation methods; and (iii) upgrade the logistics to easily transfer the rest raw materials from the vessels to the processing plants [56]. As a consequence, considerable scientific and technological efforts were recently focused on the large potential of these wastes (i.e., heads, bones, skins, and/or viscera) to be transformed into economically attractive and highly valuable by-products (as sources of lipids, proteins, peptides, collagen, chitin, vitamins, enzymes, gelatin, minerals, oils, glycosaminoglycans, polyunsaturated fatty acids, calcium, or phosphorous [57,58,59,60,61,62,63,64,65,66,67]) able to contribute to a more sustainable FPI [24,68,69,70]. In comparison to coral resources, the fish discards represent a safe, long-term sustainable source of CaP. Besides, the potential risk of disease transmission [26] to humans (i.e., bovine spongiform encelopathy, BSE) is absent. It is important to mention here that HA has a strong efficiency to store heavy metals by means of changing cations or adsorbing not changeable ones on the crystal surface [71]. Therefore, the amount of possible toxic elements in the inorganic part of fish bones should also be taken into consideration. In this respect, the concentration of heavy metals (i.e., Pb, Cd, Cu, and Hg) present in fish bones has to be below the imposed limit for inorganic bone in the case of implants used for surgical applications [72].

Biomaterials designed for bone and cartilage reconstruction should not only favor tissue regeneration at the implant site but also exhibit resistance to microbial colonization to prevent implant associated infections. These difficult-to-treat infections are caused by microbial biofilms, which grow onto the surface of medical devices and may be responsible of exacerbated or prolonged inflammation and can eventually lead to implant failure [73]. Thus, the real challenge nowadays is to fabricate implantable multifunctional biomaterials able to combine antimicrobial, anti-inflammatory, and regenerative properties. The ideal biomaterial should therefore exhibit anti-infective properties via physical-chemical features, which inhibit the initial bacterial attachment to the biomaterial or act by releasing antimicrobial agents that kill bacterial cells in the surrounding areas before approaching the surface [74]. One should also emphasize that the same requirements are essential for coatings intended to prevent nosocomial contamination of large areas in view of clinical use [52].

The aim of this review is to gather, compile, and thoroughly discuss the studies reported on the BCP materials derived from sustainable and inexpensive biogenic resources (i.e., fish discards). Thus, starting from the preparation techniques and ending with their biomedical applications, this work will tackle the transformation of fish discards into a promising and valuable alternative to synthetic materials. In addition, the development of novel BCP implant coatings with improved mechanical properties, excellent cytocompatibility, large-spectrum antimicrobial activity, and capability to promote osteogenic differentiation, collectively leading to an increased bone fixation, will also be summarized. All these characteristics could stand for an important contribution to the progress of advanced medical implants.

## 2. Biphasic Calcium Phosphate-Based Materials Derived from Fish Discards

There are reports in the dedicated literature related to the technological processing of fish discards in order to use the resulting by-products in various domains, including the biomedical one. Over the years, marine by-products were demonstrated to lead to the improvement of human health (i.e., cancer, depression, diabetes, inflammatory diseases, infections) [66,67,75]. As examples of marine by-products, one can mention the hyaluronic acid obtained from fish eyeballs [76]; collagen extracted from fish skin [77,78], swim bladders [79], or cartilaginous endoskeleton [80]; or gelatin isolated from scales [81,82,83]. Fish bones are used mostly for the production of animal feed, with a limited benefit only. In this respect, different fish species were considered: bigeye tuna (*Thunnus obesus*) [84], swordfish (*Xiphias gladius*) [13], Atlantic bluefin tuna (*Thunnus thynnus*) [13], Atlantic cod (*Gadus morhua*) [85], greater amberjack (*Seriola dumerili*) [86], European sardines (*Sardina pilchardus*) [87], sheelavati river fish (*Roho labio*) [88], barramundi (*Lates calcarifer*) [89], Atlantic horse mackerel (*Trauchurus trauchurus*), European hake (*Merluccius merluccius*), monkfish (*Lophius piscatorius*), Turbot (*Scophthalmus maximus*), or blue shark (*Prionace glauca*) [90].

### 2.1. Preparation of Fish Discards to Produce Biphasic Calcium Phosphates

**Figure 1:**
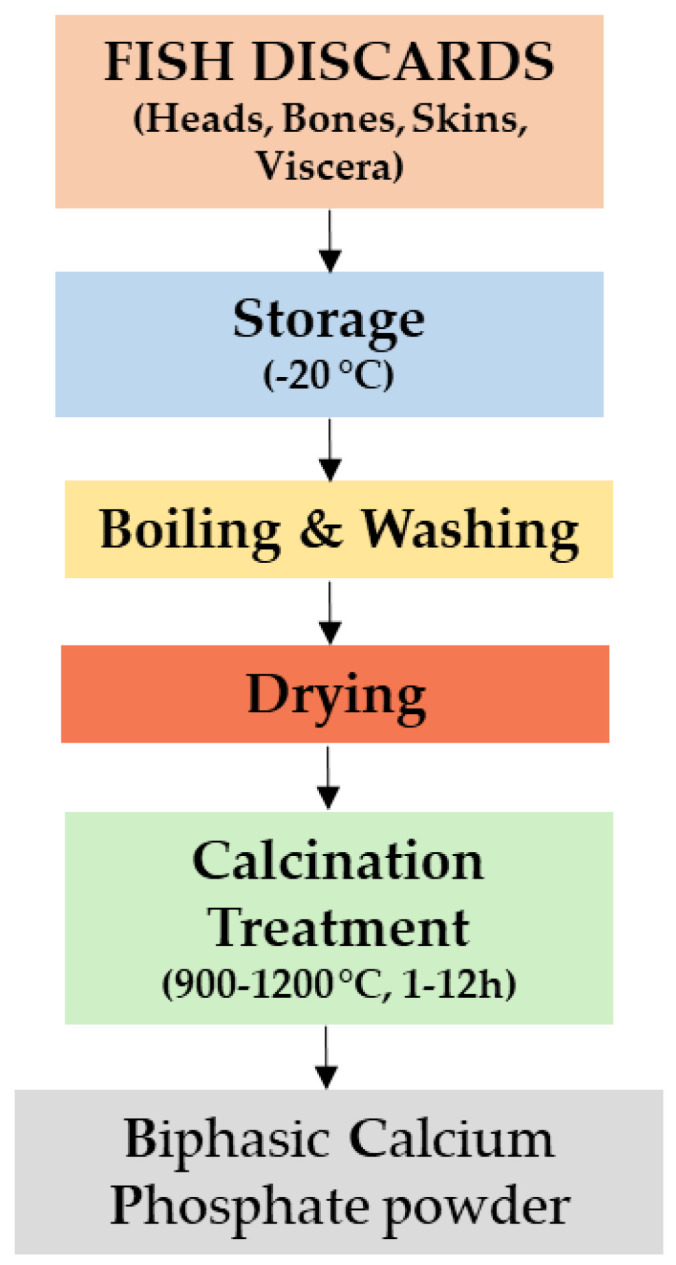
General flow diagram of biphasic calcium phosphate extraction from fish discards.

The general technological steps applied to process fish discards are presented in the flow diagram given in Figure 1.

#### 2.1.1. Pre-Treatment of Fish Discards

All reports published in the literature followed almost the same basic procedure for processing fish discards to obtain BCP materials. The first step is to store the fish discards at a temperature around −20 °C [13,85]. Next, fish discards are washed in boiling water to assure the complete removal of all organic soft impurities. This cleaning treatment is performed in different conditions.

To remove the organic substance and grease attached to their surface, fish discards were washed with distilled water [91], ethanol, and deionized water for 1 h [92], 2 h [93,94], or 3 h [32] or with boiling water [13,86,88,89,95,96,97] for 1 h [90,98,99] or 48 h [84] in an over pressurized water jet [90] and treated with 1% [98] or 5% (*w*/*v*) [91] sodium hydroxide solution and washed with ultra-pure water [98] or with 0.1 M HCl and washed with distilled water [91].

After the cleaning step, all fish discards were dried at room temperature (RT) in air [91,92,95,98] for 1 h [86,88,89] or in an oven [90] at 40 °C [97], at 55–70 °C up to 48 h [93,94], or 60 °C [91,96] for 20–30 min [32] or 72 h [99]. Next, the sterile fish discards were crushed, pulverized, and sieved in a vibratory shaker. The as-obtained powder was suspended in 40 mL distilled water under intense magnetic stirring (15 min, 450 rpm) at 70 °C. Hydrating agent (i.e., orthophosphoric acid) was added slowly to maintain a stoichiometric molar ratio of 1.5. Stirring was continued for 8 h, and the obtained slurry was dried [99].

#### 2.1.2. From Fish Discards to Powder Preparation

After the pre-treatment step, the fish discards follow a sintering process prior to being transformed into bioceramic powders.

One should note that most of the methods used to obtain HA/β-TCP biphasic mixtures are based on the chemical reaction between calcium and phosphorous, followed by a thermal treatment of the desired material [100].

**Figure 2:**
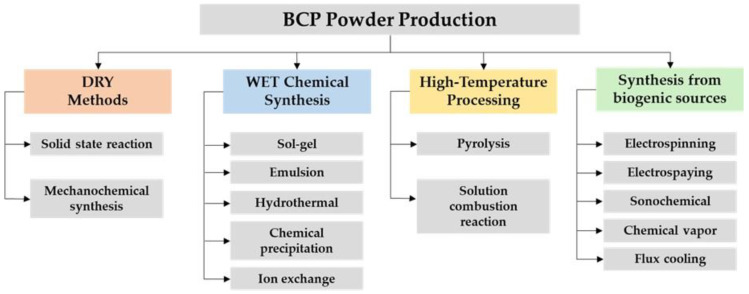
Synthesis techniques used to produce BCP powder materials.

An important number of methods were adopted by researchers to obtain BCP powders. These synthesis techniques can be generally classified into four categories, as follows: dry methods, wet chemical synthesis, high-temperature processing, and synthesis from biogenic resources (Figure 2) [101].

After a literature survey on the methods applied to produce BCP powders from fish discards, one can conclude that the most common method used for the extraction of these materials is thermal calcination [102,103,104,105] even if other methods, such as hydrothermal transformation [106], aqueous precipitation [92], laser irradiation [90], alkaline [84,107], and enzymatic hydrolysis [108], can be used as well.

In a comparative study, calcination treatments performed both at 600 and 950 °C (heating rate of 10 °C/min in air) were reported. The calcined bones were maintained at the same temperature for 12 h, consequently cooled down to 20 °C/min, and milled for 1 min. The existence of phase mixtures of HA and β-TCP was demonstrated to appear at higher temperatures [13].

In another study, fish bones were calcined at 800 °C for 3 h in an oven. After the calcination process, a centrifugal ball mill was used to produce CaP powders, with mean particle sizes of about 122 nm [98].

Another protocol mentioned that the calcination process took place at 600 °C (heating rate of 10 °C min^−1^) in air for 1 h, followed by a thermal treatment at 900 °C (heating rate of 10 °C min^−1^) in CO_2_ or in air for 1 h. This two-step sintering process under different gas atmospheres produced microporous BCPs with CO_3_^−2^ content close to the one of the human bone tissues [94].

Zhang et al. sintered fish bones in a furnace at three different temperatures of 700, 800, and 900 °C, respectively (heating rate of 5 °C/min), in air for 1 h. This study confirmed the β-TCP content in fish bones calcined at 900 °C and revealed the mechanism of changing the composition ratio of BCP by heating [32]. The same calcination temperature of 900 °C (heating rate of 5 °C/min, for 3 h) was also considered by Santosh Kumar et al. [99], who reported on the development of a polyvinyl alcohol/BCP composite for potential application as soft tissue replacement.

Piccirillo et al. [85] obtained biphasic material of HA and β-TCP from codfish bones calcined for 1 h at different temperatures (900, 950, 1000, 1100, and 1200 °C, respectively), with a heating rate of 5 °C/min. This study demonstrated that the HA/β-TCP ratio decreases with the increasing of the calcination temperature. In another work, Piccirillo et al. [100] calcined fish bones (CB) at 700 °C for 1 h (ramp of 5 °C/min). Before calcination, to increase the calcium content, a set of fish bones was pre-treated in a CaCl_2_ solution (CBCa) for 16 h at 65–70 °C. The obtained powders were milled at 200 rpm for 24 h, using as solvent isopropyl alcohol. The milled powders were dried and pressed in the form of disc-shaped pellets, which were sintered at different temperatures between 900 and 1250 °C (heating/cooling rates of 5 °C/min, dwell time of 2 h). They demonstrated that the composition of CB samples was a BCP material, whilst the one corresponding to CBCa ones was always a single phase (HA), irrespective of the applied calcination temperature.

Soumia B et al. obtained HAP/β-TCP powders from sardine fish bones after their overnight calcination in a furnace at 1000 °C in air [97].

Zhu et al. investigated the mechanism of HA/β-TCP-phase mixture formation from three type of fish species (*Salmo salar*, *Anoplopoma fimbria,* and *Sardine*) in a calcination process, which ranged from room temperature up to different high temperatures (i.e., 600, 700, 800, 900, 1000, and 1100 °C, respectively) at a heating rate of 5 °C/min in air [93].

Boutinguiza et al. [90] patented three methods to obtain BCP powders from various fish species. One of the methods is based on calcination of the fish bones at 950 °C for 12 h and the grinding of the obtained powder in a ball mill for one minute. Another method consists of the combination of calcination treatment (200–400 °C) and laser irradiation. Due to high laser energy density, the process used to obtain powders from fish bones proved to be much faster than the conventional calcination one. A shorter calcination time was obtained (from 2–3 h down to 10 min). In the third method, laser beam directly irradiated (defocused laser beam at 70 W for approximately 10 min) the blue shark bones, without samples pre-heating.

Neto et al. used cuttlefish bones for the preparation by hydrothermal transformation method (200 °C, 24 h) of undoped and doped BCP scaffolds. Bones were mixed with a phosphorous precursor solution in a proper concentration to obtain the suitable undoped BCP composition. In the case of doped scaffolds, a solution with the appropriate proportions of cationic nitrate precursor salts was also prepared and mixed with the phosphorous solution [106].

Another research group obtained BCP materials from otoliths of teleost fish (*Plagioscion squamosissimus*) using aqueous precipitation technique. The procedure was complex and consisted on several steps: (i) calcination at 1000 °C for 2 h, (ii) milling (6 min, 5000 rpm, 10 s interval), (iii) precipitation in 0.6 M orthophosphoric acid solution at 90 °C, (iv) drying in an oven at 105 °C for 24 h, and (v) calcination at 1050 °C for 3 h (heating ramp of 2 °C/min). The scope of this research was to evaluate the properties of BCP materials depending on the pH solution and precipitation time conditions at RT (48 h using pH values of 4, 5, and 6 and 96 h with pH values of 6, 8, and 10, respectively) [92].

By alkaline hydrolysis, Fernández-Arias et al. [109] obtained BCP from whole individuals of fish discards derived from aquaculture processing. The hydrolysis process was followed by a calcination treatment in a furnace at temperatures of 750 °C and 950 °C for 10 h (heating rate of 10 °C/min).

The combination of natural CaPs, in general, and BCP, in particular, with other materials, in terms of biocompatible alloys, ceramics, and polymers, represents an alternative way towards the development of new biomaterials with improved performance and mechanical responses with the aim to mimic those of natural bones and tissues [95,105,110].

Kiyochi Junior et al. calcined fish bones at 900 °C for 8 h. The resulting material was grounded for 8 h at 300 rpm. To minimize particle’s aggregation, the as-obtained powders were sonicated for five minutes in a bath containing acetone. BCP powders were mixed with Nb_2_O_5_ (1:1 ratio of volume percent) and milled for 3 h at 300 rpm. Nb_2_O_5_ was chosen due to its biocompatibility and high resistance to corrosion [111]. NbBCP nanocomposite was pressed as discs (8-mm diameter, 1-mm thickness) and sintered in air atmosphere at 1000 °C for 1 h. After sintering, the discs were ultrasonically washed with acetone, water, and alcohol and autoclaved [95].

Silva et al. [105] obtained BCP powders from Pintado fish bones (*Pseudoplatystoma corruscans*) after applying a calcination temperature of 900 °C for 8 h. The resulted products were milled for 8 h at 300 rpm [104]. The as-obtained BCP powder was combined with a piezoelectric polymer, polyvinylidene fluoride (PVDF), which is characterized by a high proliferation of osteoblast cells [112]. The composite powder was homogenized, compacted under uniaxial pressure of 114 MPa, and sintered at 170 °C in air for 1 h. The same combination, BCP+PVDF, following a similar protocol, was studied by Bonadio et al. [110], who demonstrated the improvement of the bioactivity and consequently their potential use for bone-tissue engineering.

In conclusion, the most applied method to produce BCP powder materials is calcination, using high temperatures of generally over 900 °C.

### 2.2. The Use of BCPs

BCP materials, pure or modified, obtained from fish discards by the aforementioned methods were used in different forms, such as powders, coatings, scaffolds, and nanorods, depending on the envisaged application.

#### 2.2.1. BCP as Coatings

BCP ceramics of biogenic origin can be transferred onto various type of substrates using a variety of methods. Up to date, most of the reported results referred to pulsed laser deposition (PLD) [113] and radio frequency-magnetron sputtering (rf-MS) [40,114].

In the field of coatings growth, the PLD technique stands as a simple, versatile, and fast-processing method that allows for a precise control over the deposition rate and morphology to obtain high-quality films [115,116]. In particular, for the synthesis of bioceramic materials, one of the major advantages of PLD is its capability to grow stoichiometric films (due to the high ablation rate, all elements are evaporated at the same time) [117].

BCP coatings were synthesized by PLD using a pulsed UV laser source from bioceramic targets of sea bream (*Sparus aurata)* and salmon (*Salmo salar)* fish bones. Within this research, bone implant coatings with both biocompatible and antimicrobial behaviors were fabricated [113].

Rf-MS is a physical vapor deposition method capable of generating uniform, dense, thin, low-impurity, and adherent coatings with desired crystallinity [118].

Behera et al. [40] obtained BCP coatings from raw fish scales by rf-MS onto Ti-6Al-4V substrate. They studied the properties of three sets of BCP coatings, varying the deposition time (4, 6, and 8 h, respectively) at RT. The reported results evidenced similar proportions of HA and β-TCP for all synthesized BCP films. In another study, the same team investigated the effect of surface roughness and chemistry on the different physical and biological performances of BCP coatings synthesized by rf-MS onto laser surface textured Ti6Al4V [114]. The obtained results showed an increase by ~200% of the initial roughness of bare substrates as compared to laser micro-textured ones. A significant enhancement in the wetting behavior due to both laser texturing and deposition of BCP films was indicated. Consequently, protein adsorption and a higher number of well-spread MG63 osteoblast cells were also observed.

#### 2.2.2. BCP as Scaffolds

BCP porous scaffolds obtained from cuttlefish (*Sepia officinalis*) powders undoped and doped with Sr^2+^, Mg^2+^, and/or Zn^2+^ ions were sintered and then coated with poly(ε-caprolactone) (PCL), poly(DL-lactide) (PDLA), poly(ester amide) (PEA), or poly(ester urea) (PEU) to improve their compressive strength [106]. The scaffolds were dipped in the polymer solutions under partial vacuum for approximately 5 min, dried overnight, and kept at RT for seven days to eliminate any remnant organic solvent. In another study, the same type of BCP porous scaffolds were functionalized by immersion in Sr-, Mg-, and Zn-doped sol-gel-derived BG solutions. The scaffolds were kept in a vacuum chamber under a pressure of 0.4 bar for 20 min and dried in an oven at 100 °C for 24 h. For comparison reason only, uncoated scaffolds sintered at 700 °C (heating rate of 0.3 °C min^−1^) for 2 h [119] were also investigated.

#### 2.2.3. BCP as Nanorods

One-dimensional, two-phase mixture BCP nanorods were synthesized from cuttlefish bone through the mechanochemical method and blended with polyvinyl alcohol (PVA) to develop a PVA/BCP composite hydrogel. The blending of PVA with the BCP nanorods produced a reinforced material owing to the high aspect ratio, characterized by the HA stability and β-TCP reactivity [99,120].

A literature summary regarding the preparation of BCP materials from various fish discards is presented in Table 1.

## 3. Properties of Biphasic Calcium Phosphate Materials

BCP materials derived from abundant, sustainable resources, like fish discards, are yet under investigation to evidence their beneficial effects, especially in domains with impact on human health. In this respect, for their evaluation, many complex investigation methods are used. Morphological, structural, mechanical, and functional properties of BCP materials obtained from fish discards are presented in the following sections.

### 3.1. BCP Powders

#### 3.1.1. Morphological Characteristics

**Figure 3:**
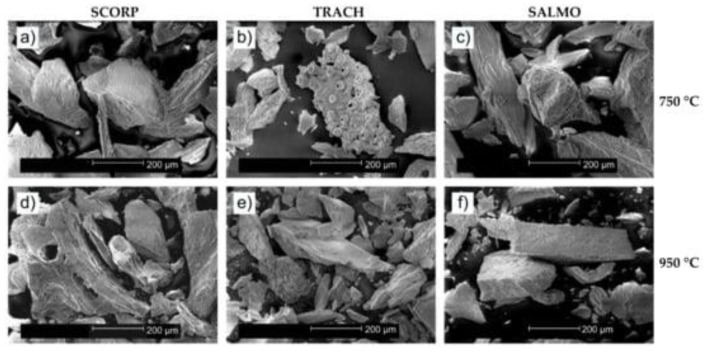
SEM images of porous and irregular structures in case of BCP powders derived from Red scorpionfish (SCORP) bones calcined at (**a**) 750 °C and (**d**) 950 °C, Atlantic horse mackerel (TRACH) bones calcined at (**b**) 750 °C and (**e**) 950 °C, and salmon (SALMO) bones calcined at (**c**) 750 °C and (**f**) 950 °C. (Magnification bar: 200 µm). Reproduced with permission from [109]. Copyright MDPI, 2021.

Results from the literature on BCP powders evidenced different morphological features. SEM images of BCP powders derived from *Scorpaena scrofa*, *Trachurus*, and *Salmo salar* fish bones calcined at two temperatures (750 and 950 °C, respectively) revealed the same porous-shaped structure in both cases. In addition, granules of irregular morphologies with a porous structure were also observed (Figure 3) [109].

In another study, SEM images of BCP powders obtained from cod fish bones annealed at 1000 °C presented a morphology both with round grains (with dimensions of 300–500 nm) and with much larger and more crystalline-looking ones (crystals with a needle-like aspect, 500-nm wide and several microns long). Samples immersed in CaCl_2_ solutions showed a different morphology as compared to untreated ones. Thus, only round, poorly crystalline grains were observed, whilst the elongated ones were absent. At a higher magnification, the powder morphology had a mottled or cracked appearance with equally sized and closely packed rounded features (diameter of 30–50 nm), which were not observed in the untreated samples [85]. As reported in literature, the morphology with an oriented grain structure could have positive effects on the mechanical, dielectric, piezoelectric, and biomedical properties [123].

SEM micrographs corresponding to the powders obtained from grey triggerfish (GT) skin and black scabbardfish (BS) bones treated above 600 °C evidenced the increase of particles size with the calcination temperature. The GT powders treated at 600 °C consisted of elongated, rod-like crystals (200–400-nm length, 100-nm width). At 800 °C, a secondary flower-like morphology was evidenced and attributed to rhenanite crystals, known to enhance osteogenesis when applied as bone substitutes. The powder from BS bones treated at 600 °C is made of crystals with diameters smaller than 100 nm [121]. Another effect of the increase of calcination temperature was the decrease of their surface specific area (SSA) for both BS bones and GT skin. After calcination at 400 °C, the powder obtained from GT skin was characterized by a SSA_BET_ of 110.42 m^2^g^−1^, which decreased to 2.10 m^2^g^−1^ for powders obtained at 1000 °C. In the case of BS bones, SSA_BET_ decreased from 133.47 m^2^g^−1^ (400 °C) to 2.53 m^2^g^−1^ (1000 °C). It was shown that the SSA_BET_ value decreased with the increase of the particle size [121].

On the other hand, a decrease in particle size was obtained for otoliths of teleost fish (*Plagioscion squamosissimus*) synthesized at different precipitation times and high pH values (8–10) [92].

**Figure 4:**
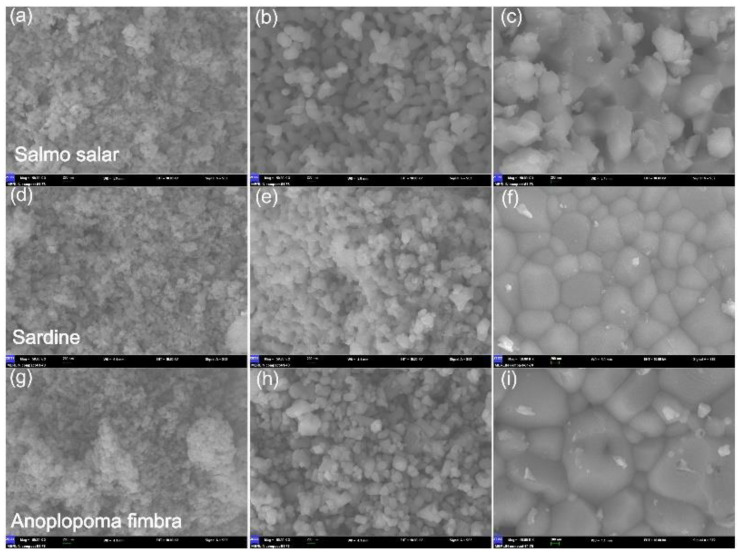
SEM images of BCP powders produced from fishbones at different calcination temperatures: *Salmo salar* at 700 °C (**a**), 900 °C (**b**), and (**c**) 1100 °C; sardine at 700 °C (**d**), 900 °C (**e**), and (**f**) 1100 °C; and *Anoplopoma fimbria* at 700 °C (**g**), 900 °C (**h**), and (**i**) 1100 °C. (Magnification bar: 200 nm). Reproduced with permission from [93]. Copyright Elsevier, 2017.

Zhu et al. investigated the morphology of the BCP powders depending on the applied calcination temperature. At 700 °C, the BCP grains had a short, rod shape, with sizes in the 30–100-nm domain. At this temperature, a porous structure between grains was not visible. A higher calcination temperature (i.e., 900 °C) promoted bigger particles (50–200 nm) and a porous structure homogeneously distributed between them. When increasing the calcination temperature (1100 °C), SEM micrographs revealed that the particle size augmented, and the great majority of the small particles clustered into bigger ones (200 nm), which generated a dense microstructure. Temperatures higher than 1000 °C determined the increase of the crystallinity degree and particles’ size. In turn, this affected the degradation rate (Figure 4) [93].

**Figure 5:**
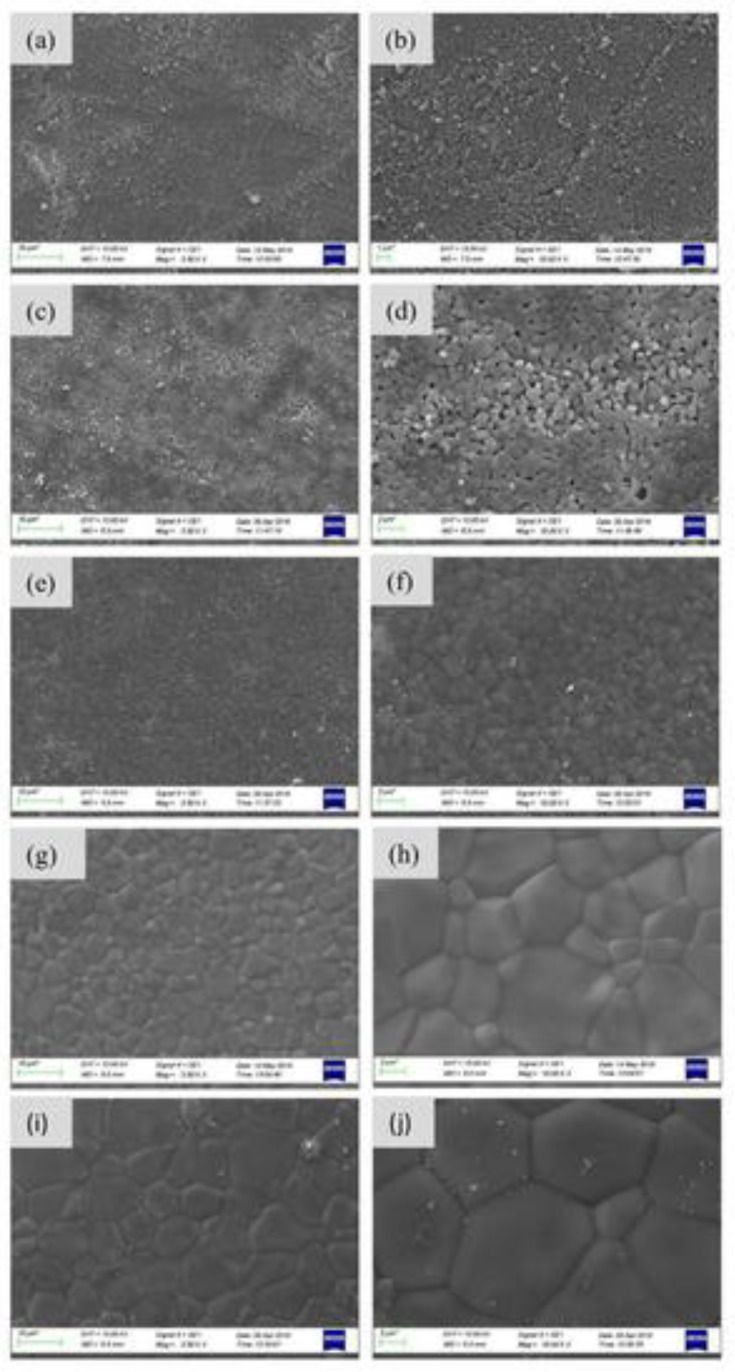
SEM images of porous structures in case of BCP powder derived from salmon fish bones at different sintering temperatures: (**a**,**b**) 800 °C, (**c**,**d**) 1000 °C, (**e**,**f**) 1100 °C, (**g**,**h**) 1200 °C, and (**i**,**j**) 1300 °C. (Magnification bars: 10 µm (left column), 2 µm (right column). Reproduced with permission from [98]. Copyright MDPI, 2020.

Similar morphological and particle size modifications were observed by Bas et al. [98] for the BCP powder obtained from salmon fish bones sintered at temperatures in the (800–1300) °C range (Figure 5). At 800 and 1000 °C, a porous structure between the grains was visible. At higher sintering temperatures (1200 and 1300 °C), the SEM investigations showed an increase of sample’s density, a reduction of the intergranular porosity, and the growth of large grain agglomerates.

**Figure 6:**
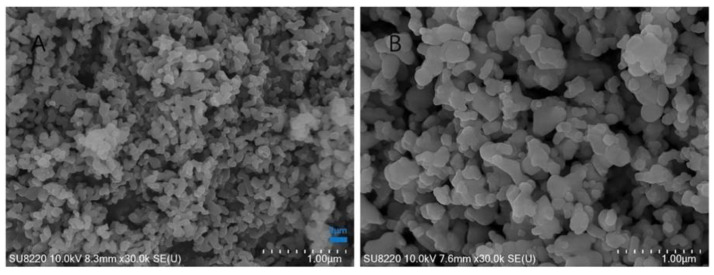
SEM images of porous structures in case of (**A**) untreated and (**B**) CO_2_-treated BCP powders derived from salmon fish bones. Pore dimensions: (0.03–0.15 µm). (Magnification bar: 1 µm). Reproduced with permission from [94]. Copyright IOPScience, 2020.

Dou et al. investigated the microporous CO_3_^2−^-containing BCP ceramic from *Salmo salar* bone in a CO_2_ atmosphere in comparison with pure BCP bioceramic material. SEM images showed an interconnected natural macroporous structures ranging from 0.1 to 3 µm for both BCP products. The particle sizes of pure BCP powder were significantly larger than those of the CO_2_-BCP one (Figure 6).

The pore size distribution and porosity of the products were also evaluated. The measurements estimated that both structure of powders have a pore size in the (0.03–0.15) μm interval and that CO_2_-BCP has a sharper peak than pure BCP. This indicated that the CO_2_-BCP powder is characterized by a higher density of micropores [94]. According to the authors, the addition of CO_3_^2−^ into BCP influenced the morphology of the powder, which further determined the decrease of crystallinity degree, the appearance of a large number of micropores, and the increase of SSA.

In another study, the powder obtained from swordfish (*Xiphias gladius*) and tuna (*Thunnus thynnus*) at different calcination temperatures (600 and 950 °C) exhibited a morphology composed of rod-like shape particles with submicron average size in crystalline structures having lattice parameter of ~0.8 nm [13].

The morphology of the powder obtained after the thermal treatment at 900 °C of Hairtails bones consisted of a structure with pores uniformly distributed. This porous structure facilitates the body fluid flow or the adherence and proliferation of cells and tissues. In addition, SEM investigations evidenced the shape of the two components of BCP, i.e., rod-shaped HA particles and round β-TCP ones (5–10 µm diameter) [32].

**Figure 7:**
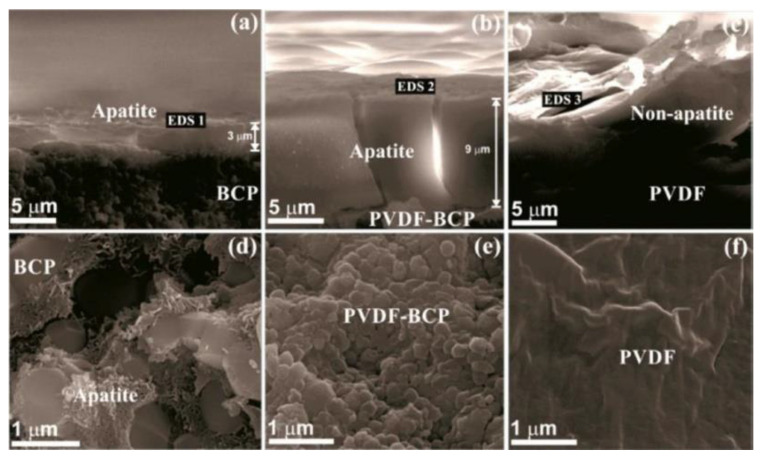
Cross-sectional and top view SEM images of (**a**,**d**) BCP, (**b**,**e**) PVDF-BCP, and (**c**,**f**) PVDF. Reproduced with permission from [105]. Copyright SciELO, 2018.

Silva et al. [105] reported on the morphological evaluation of BCP pure and mixed with PVDF after immersion in simulated body fluid (SBF) for seven days. The BCP samples revealed a highly porous morphology, which can lead to a faster osteointegration [124,125]. The most important observation was the growth of a HA layer on the surface or inside the pores, which improves the bioactivity of the material. For pure BCP samples, the thickness of the HA layer was estimated at ~3 µm while for PVDF-BCP composite at ~9 μm (Figure 7). In addition, on the surface of the PVDF-BCP composite, spheres with ~10 μm in diameter were observed, which promote the formation of a uniform HA layer [105,126].

On the other hand, Bonadio et al. [110] compared the morphologies for PVDF and BCP-PVDF samples before and after immersion in SBF (for seven days). PVDF and BCP-PVDF un-immersed composite showed almost the same morphologies. The surface and fractured surface of composites immersed in SBF revealed morphological and microstructural changes. This was due to the globular nucleation of an apatite layer (~1.45 µm) composed of agglomerated nanometric grains (~3.30 µm in diameter).

The BCP morphology was modified also when powder was mixed with others ceramics, such as Nb_2_O_5_. The grains’ dimensions increased and the shape became rod-like after the mixing of the two materials. Rounded grains with size distribution of 0.5 and 1 μm were observed for pure BCP powders.

In case of NbBCP, the obtained grains had a rod-like shape, with sizes in the (1.0–2.5) μm range. In addition, SEM analysis evidenced the existence of a less porous microstructure for NbBCP [95].

#### 3.1.2. Elemental Composition

Many studies performed on BCP materials revealed that chemical and phase compositions have direct consequences on their performance to fit the purpose of medical applications.

In general, the calcium deficiency determines the HA/β-TCP ratio in the BCP materials. Different HA/β-TCP ratios have been investigated to enhance BCP properties. An ideal balance between these two phases may improve mechanical properties and increase the biological response of BCP materials. From a biological point of view, the difference in composition ratio of each phase (HA/β-TCP) has a crucial role in the in vivo performance, such as biodegradation, precipitation of apatite crystal, protein adsorption, and cellular behavior [127].

The qualitative evaluation of the chemical composition of BCP powders obtained from red scorpionfish (*Scorpaena scrofa*), Atlantic horse mackerel (*Trachurus trachurus*), and salmon (*Salmo salar*) discards performed by EDS evidenced the presence of the main components: P, Ca, Mg, and Na. To determine Ca/P molar ratio, the quantitative evaluation of the chemical composition of the obtained powders was performed by inductively coupled plasma optical emission spectroscopy (ICP-OES). For the three fish species, the values of Ca/P molar ratio were found to decrease with the increase of the calcination temperature. For example, in the case of powder obtained from red scorpionfish (*Scorpaena scrofa*), the Ca/P ratio was 1.77 (at 750 °C) and 1.75 (at 950 °C). These values are, however, greater than that of stoichiometric HA (1.67) due to the substitution of (PO_4_)^3−^ groups by CO_3_^2−^ [109]. The decreasing of the Ca/P ratio in all three cases with the increase of the calcination temperature could be attributed to a partial transformation of the HA phase in β-TCP and, consequently, to the formation of BCP compound. The ICP-OES technique was also used to detect the presence of heavy metals in BCP powder, knowing the ability of HA to store them by means of ionic substitutions. Heavy metals (like Hg, Cd, and Pb), which are present in fishes, should be avoided in any products dedicated to humans because of their harmful effects, such as toxic or carcinogenic behavior. For the three fish species, the authors found out that the amount of these elements present in BCP powder was lower than the maximum permitted value for organic bone implant materials (Cd: 0.68 ppm, Pb: 1.6 ppm, Hg: 0.04 ppm) [109].

Piccirillo et al., in their study regarding BCP materials obtained from cod fish bones, reported on the effect of thermal treatment at different high temperatures (900–1200 °C). This determined a composition close to the optimal value of 75:25 in all samples. Among them, those annealed at 950 and 1000 °C had the highest HA content, 72.6 and 71.7, respectively. In case of powder annealed at 1000 °C, the elemental analyses evidenced the presence of sodium, chlorine, and fluorine ions, which have many benefits for the use of this material in medicine. It is known that a high fluorine concentration can accelerate the bone osteointegration [128], whilst chlorine and sodium ions could positively affect the resorbability. Elemental analysis of these samples showed that the Ca/P ratio had a value of 1.49, which is smaller than the one corresponding to stoichiometric HA (Ca/P = 1.67). This result could be related to the formation of β-TCP (Ca/P = 1.5) phase in the material [85].

Chemical treatments of calcium-containing compounds were performed to adjust the Ca/P ratio. CaCl_2_·2H_2_O, Ca(C_2_H_3_O_2_)_2_, and NaF salt solutions were used. In the case of CaCl_2_·2H_2_O and Ca(C_2_H_3_O_2_)_2_ solutions, a growth of calcium concentration and a decrease of the phosphorus concentration were observed, with the Ca/P ratio around the stoichiometric value. This can be reflected in the increase of HA percentage in the biphasic material. In case of chemical treatment with NaF solution for different periods of time (3, 6, and 16 h, respectively), the authors reported Ca/P values very different depending on the stirring duration, from 1.45 for 3 h up to 2.01 for the longer period of time. The higher values of Ca/P ratio were caused by the decrease of the P concentration [85].

The BCP powders obtained from GT skin and BS bones have in composition Ca and P, predominantly, and traces of Mg and Sr, which are known to enhance osteogenesis when used as bone substitutes. Quantitative estimation of Ca, P, Mg, and Sr content was performed by ICP-OES. Similar to other reports, Ideia et al. [121] concluded that the composition of BCP powders (ratio between HA and β-TCP) from fish discards depends on the calcination temperature. Powders obtained from the calcination of GT skin have a Ca/P molar ratio in the range of (1.65–1.62) for the calcination temperatures from 400 to 1000 °C, slightly lower than for stoichiometric HA. BS bone-derived powders have a different chemical composition with respect to those extracted from GT skin. In this case, the values for Ca/P molar ratios were situated in (1.47–1.54) domain for the same temperatures, which are significantly lower than the standard one.

The similar tendency was reported by Bas et al. [98] in their study about the BCP powder obtained from salmon fish bones calcined at temperatures between 1000 to 1300 °C. They performed the composition investigations by EDX and found that the powders mainly contained Ca, P, O, and Mg. The powder sintered at 1100 °C had a Ca/P ratio of 1.67, whilst the one treated at a higher temperature (1300 °C) was characterized by a smaller ratio (Ca/P = 1.52). In addition, the presence of Mg ions along with other trace elements present in the salmon fish bone may improve the desired osteogenic properties [129].

The Ca/P molar ratios of the powders obtained from *Salmo salar*, *Anoplopoma fimbria,* and sardine fish bones calcined at 900 °C and reported by Zhu et al. varied between 1.5 and 1.67. This clearly demonstrated that the compounds contained both HA and β-TCP phases. Among the three powders, the Ca/P molar ratio of *Salmo salar* had the minimum value (1.5), whilst *Anoplopoma fimbria* had the maximum (1.67). As expected, the Ca/P ratio is influenced also by the fish species. For example, the highest content of β-TCP was found in powder derived from *Salmo salar* bones, and the smallest one in the powder derived from *Anoplopoma fimbria* [93]. The X-ray fluorescence (XRF) and ICP-OES analyses evidenced the presence of mineral elements (Mg, Na, K, and Sr) in variable concentration depending on the life environment for every species.

An average molar ratio (Ca/P ~ 1.87) was determined for powders obtained from sword- and tuna fishes. This high value compared to that of the stoichiometric HA was attributed to the existence of carbonate ions, which substituted the phosphate ones. Na, Mg, K, and Sr were also present in trace amounts. Besides these elements, the occurrence of heavy metals in the obtained powders from fish bones was detected, fortunately, below the allowed limit (Pb: 0.7 mg/kg, Cd: 0.9 mg/kg, Hg: 0.1 mg/kg) [13].

#### 3.1.3. Structural Characteristics

For the crystallography and phase identification and quantification of BCP materials the most frequently applied method is XRD. From literature, it is noticed that in case of BCP, the usual XRD scan range is from 20° to 60° because most of the CaP phases have the strongest peaks in this domain [130]. By XRD analysis, the HA/β-TCP ratio in the BCP could be evaluated. This represents the ratio of intensities between the most intense diffraction peaks of the HA and β-TCP phases [131].

The results on chemical characterization of BCP major functional groups reported in the literature were mostly obtained by Fourier-transform infrared spectroscopy (FTIR).

In the following, structural properties of BCP powders obtained from fish discards calcined at different temperatures are reviewed. Information to be highlighted are the crystallinity of BCP powders and the existence of specific intensity peaks of HA and β-TCP, respectively.

ICP-OES results obtained on red scorpionfish (*Scorpaena scrofa*), Atlantic horse mackerel (*Trachurus trachurus*), and salmon (*Salmo salar*) discards were in good agreement with the XRD investigations. The main peaks identified were slightly shifted with respect to synthetic HA, which indicated a change in the unit cell parameters. This modification of lattice parameters is determined by the substitutions of Ca ions with Na, Mg, Sr, or K ones [109].

Except for the Salmon powder, the crystallite size of the other powders calcined at 750 °C was around 50 nm. This value is similar to the average crystallite size of mature bone [132]. In the case of calcination at high temperatures (i.e., 950 °C), an increase in crystallinity, and a growth of the crystallite sizes to values around 60 nm were observed [109].

FTIR investigations, in agreement with the XRD results, revealed that the spectra recorded for the three fish powders mentioned above exhibited the existence of narrow bands, which suggested a high degree of crystallinity. Additionally, phosphate groups were identified at ~960 cm^−1^ and between (1000–1200) cm^−1^ for the three species as a confirmation of the presence of β-TCP phase [109].

The XRD patterns of the powder derived from salmon fish bones calcined at 800 °C and sintered, as pellets, from 1000 to 1300 °C showed the presence of crystalline phases (i.e, HA and TCP). The TCP phase increased, whilst the amount of the HA one decreased with the temperature. Similar to other reports, when the sintering temperature reached the highest value (i.e., 1300 °C), the powder became a mixture of HA and TCP phases [98]. FTIR analyses exhibited the characteristic HA bands, peaking at 560, 597, 629, 960, 1011, and 1084 cm^−1^, respectively. With the increase of the sintering temperature (around 1100 °C), the CO_3_^2−^ functional group disappeared, and the TCP was visible [98].

The crystallinity of the powders obtained from salmon fish bones and chemically treated in CO_2_ dynamic flux presented some changes. The XRD patterns revealed that the degree of crystallization of BCP in CO_2_ was lower than of BCP in air. This behavior was caused by the substitution of CO_3_^2−^ functional groups, which promotes the lattice distortion. Consequently, incorporation of CO_3_^2−^ affects the crystal structure of HA, and the *a*-parameters decreased from 9.48 nm to 9.29 nm [94]. The presence of characteristic peaks of β-TCP at 1122 cm^−1^ and 959 cm^−1^ for both untreated and chemically treated BCP was detected by FTIR. The powder in CO_2_ was characterized by high-intensity bands at 1465 cm^−1^ and 875 cm^−1^, which were attributed to CO_3_^2−^. The peaks of (PO_4_)^3−^ functional group (1100–900 cm^−1^) had a sharp shape for untreated BCP and a wide one for the chemically treated BCP [94].

The XRD investigations of the powders derived from cod fish bones calcined at temperatures in the range of (900–1200 °C) showed the presence of both HA and β-TCP phases [85]. The FTIR investigation of the powders obtained at 1000 °C confirmed the biphasic nature of this material with peaks belonging to the HA phosphate ions at 1092, 1046, 980, 962, 601, 568, and 473 cm^−1^, respectively, and characteristic ones for β-TCP at 1122 cm^−1^ [133].

Picirilo and co-workers [85] reported that the treatments in CaCl_2_·2H_2_O and Ca(C_2_H_3_O_2_)_2_ salts solution affected the microstructure of the BCP powders derived from fish discards (as visible from SEM micrographs) and conducted to a well-sintered product with oriented, elongated grains. At nanoscale, the more crystalline-looking treated samples had also an unusual, mottled surface when annealed at 1000 °C.

The XRD pattern of the sample treated with CaCl_2_ solution at 75 °C for 16 h evidenced the detection of HA diffraction peaks only. This result was confirmed by IR analyses where the peak at 1122 cm^−1^, associated to β-TCP, was not detected. This was the CaCl_2_ solution effect on the HA/β-TCP ratio: the percentage of HA increasing with longer periods of time in solution at 75 °C. However, in case of CaCl_2_ chemical treatment at RT, the biphasic material was determined in a ratio of 85:15 (HA:β-TCP). The presence of biphasic material was detected also for the samples treated with Ca(C_2_H_3_O_2_)_2_ solution at 75 °C, with a HA composition percentage of 92.8 for 16 h of stirring. In those cases, the increase in the calcium concentration could be attributed to a high pH value of the solutions caused by the alkaline nature of the acetate ion [85,134]. On the other hand, the phosphorus concentration decreased for longer chemical treatments, probably due to the slow dissolution process of the HA-based material [135]. The same authors reported later [100] the effects of the sintering process (at 900, 1000, 1100, 1200, and 1250 °C, respectively) on the sample’s composition. The powders derived from cod fish bones consisted of 73.2 wt.% HA and 27.8 wt.% β-TCP, while those treated in CaCl_2_ contained pure HA only. The most relevant result was that the amount of β-TCP slightly increased with the temperature in the case of untreated powders. The XRD investigation revealed that both HA and β-TCP phases were detected in all cod bone samples and no significant variation in the composition ratio of HA:β-TCP (75:25) with the sintering temperature was evidenced. The treatment of the BCP powders in CaCl_2_ solution for different time intervals determined a total weight loss of ~40%, which is in agreement with the data reported in the literature [136]. A first weight loss, related to the release of the absorbed water, arose at temperatures below 200 °C. Higher weight losses were observed for 200 °C < T < 500 °C. Two peaks were visible: (i) a small one, which corresponded to the release of residual organics and water present in the lattice structure, and (ii) a large one, which was attributed to the release of organic matter [137]. These results were confirmed by the DTA investigations, with the appearance of two exothermic peaks corresponding to these two weight losses. For temperatures higher than 850 °C, an endothermic heat exchange was revealed and related to the lattice rearrangements and apatite crystallization [85]. An important weight loss was also observed for temperatures lower than 600 °C. However, a smaller loss was indicated for temperatures higher than 800 °C due to the release of the chloride ions from the lattice.

The XRD patterns recorded for powders derived from GT skin showed that the main component phase is attributed to HA and characterized by intense and sharper peaks. It was also demonstrated that the composition percentage of HA was in direct dependence of the calcination temperature. This revealed an increase of the structural order and crystal growth with the calcination temperature. For the material obtained at 400 °C, the HA structure was characterized by very broad and poorly defined diffraction peaks. Besides HA, other phases, like halite (Hal, NaCl), with low-intensity peaks, were identified. The resulted material was composed of poorly crystalline HA (~95 wt.%) and Hal (~5 wt.%) [121].

Besides HA and Hal phases, XRD patterns of samples obtained at 600 and 800 °C revealed the existence of other phases with traces of rhenanite (Rhe, NaCaPO_4_). In addition, in the powder calcined at 600 °C, the presence of β-TCP phase was also confirmed. In case of thermal treatment at 1000 °C, HA was the majority phase, with small amounts of MgO_2_ and cubic trisodium phosphate (γ-Na_3_PO_4_). From the crystalline point of view, GT skin samples treated at three temperatures (600, 800, and 1000 °C, respectively) presented narrow and well-defined peaks, which were comparable to commercial HA. A similar structure was also observed in the case of BS bones treated at 600, 800, and 1000 °C, respectively [121].

The XRD patterns of samples derived from BS bones calcined at (600, 800, and 1000 °C, respectively) pointed out the existence of HA and β-TCP peaks, whilst at 400 °C, only HA appeared. The β-TCP composition percentage increased with the calcination temperature, from 17.5 wt.% at 600 °C up to 40.1 wt.% at 1000 °C [121]. The FTIR analyses confirmed the XRD results. The existence of HA was demonstrated by the presence of small peaks at 870–880 cm^−1^ and 1400–1500 cm^−1^ [138]. For the TF skin samples treated at 600 °C, and for those of SF bones treated at 600, 800, and 1000 °C, peaks at 947, 978, and 1119 cm^−1^ were detected. These were characteristic to β-TCP phase. It is worth noting that the peaks’ intensities increase with the calcination temperature, which was in good agreement with the XRD results [121].

A similar trend regarding the crystallinity of HA obtained from *Salmo salar*, *Anoplopoma fimbria*, and sardine bones was reported by Zhu et al. [93]. In case of raw fish bones, the XRD patterns evidenced the presence of monocrystalline apatite, characterized by wide and small diffraction peaks, which indicated the existence of small-sized grains and a low crystallinity degree of HA. The effects of the thermal treatments on the HA structure were reflected in the (i) presence of narrow and sharp diffraction peaks, (ii) increase of the crystallinity degree, and (iii) crystals’ growth during high-temperature calcination. At temperatures above 700 °C, XRD patterns confirmed the appearance of β-TCP diffraction peaks with intensities depending on the calcination conditions. The content of β-TCP in the powders obtained from three fish species showed a tendency to increase with the applied temperature [93]. In the same study, the authors reported on the behavior of the main functional groups, evaluated by FTIR, before and after samples’ calcination. For fish bones calcined from 700 to 1100 °C, the presence of the (PO_4_)^3−^ and the OH^-^ peaks was visible. The peaks situated at 984 cm^−1^ and 1121 cm^−1^ were attributed to β-TCP, which confirms the nature of BCP materials produced from calcined fish bones [93]. To study the thermal stability of the bones, they were placed in a thermogravimetric analyzer and heated from RT to 1000 °C. TGA analyses revealed the similar weight changes of the three types of bones. The first weight loss was for T < 200 °C, with a maximum change at 65–75 °C, which corresponded to the loss of absorbed water in fish bones. The second occurred at 200 °C < T < 600 °C, when the water is removed from powder, and the collagen, protein, and grease are partially combusted to release CO_2_ and H_2_O. For T > 700 °C, the rate of weight loss in fish bones was 6.32% for *Salmo salar*, 5.43% for sardine, and 5.25% for *Anoplopoma fimbria*. Around 1000 °C, the mass of *Salmo salar*, sardine, and *Anoplopoma fimbria* was 47.89, 53.61, and 39.31%, respectively [93].

The balance between HA and β-TCP phases in BCP materials obtained from otoliths of teleost synthesized at different pH values was evaluated by XRD [92]. The diffraction analysis evidenced that, at low pH values (4, 5, 6), HA and β-TCP phases were formed, whereas a single-crystalline phase of nano-sized HA appeared at high pH values (6, 8, 10) even after 96 h of precipitation time. The amount of crystalline HA increased with the pH value for higher precipitation times. The presence of the two phases, β-TCP and HA, was also confirmed by FTIR analysis [92].

Boutinguiza et al. [13] reported on CaPs obtained from sword- (*Xiphias gladius*) and tuna (*Thunnus thynnus*) fish bones with a similar tendency to form a biphasic compound, depending on the calcination temperature. At 600 °C, the powder was composed of HA phase only, with well-defined peaks for both fish species. At elevated temperatures (i.e., 950 °C), the presence of β-TCP apart from HA was detected, with an estimated concentration of 13 wt.% for swordfish bones.

The obtained powders were composed of crystalline phases, with crystallite size around 50 nm for the powders calcined at 600 °C. In addition, the crystallite size increased (up to 67 nm) with the calcination temperature (i.e., 950 °C) due to the long post-calcination treatment time (12 h). Fish bones heated at 600 °C better preserved the inherited properties from raw materials than those calcined at 950 °C [13]. The FTIR spectra of all samples exhibited narrow bands, which suggested a high degree of crystallization. The characteristic bands for (PO_4_)^3−^ group were present [13]. The XRD and FTIR results were sustained, also, by Raman investigations, which stated that at high heating temperatures (i.e., 950 °C), partial transformation of HA to β-TCP, and formation of biphasic powder from fish discards is possible [13].

In case of powders obtained from Hairtails fish bones, the general trend was preserved in terms of dependency on the calcination temperature. With the increase of the temperature from 700 to 900 °C, the crystallinity decreased for HA and increased for β-TCP. This is an effect of decomposition at 700 °C of the Ca-deficient HA to HA and β-TCP [3,88,139,140]. The absorption peaks characteristic to phosphate ions were detected at 1092, 1046, 980, 962, 601, 568, and 473 cm^−1^ and for β-TCP at 1122 cm^−1^, which confirmed the formation of BCP powder at high temperatures (900 °C) [32].

The well-homogenized BCP-PVDF mixture was characterized, from the structural point of view, by the overlapping of the dominant and very crystallized BCP with the amorphous PVDF patterns. For BCP ceramic, the diffraction patterns of HA and β-TCP phases were present. In case of PVDF sample, the XRD pattern showed an amorphous structure over which two other crystalline ones were superimposed. These crystalline structures were attributed to the nonpolar (α) and polar (β) PVDF polymorphic phases [105].

Bonadio et al. [110] compared the XRD patterns of the BCP-PVDF before and after SBF immersion and reported on the existence of an apatite layer on the surface of the analyzed compound.

The FTIR investigation detected the presence of (PO_4_)^3−^ and (OH^−^) groups characteristic to HA and β-TCP in BCP. The absorbance spectrum of the BCP-PVDF was composed by the same bands of pure BCP and PVDF.

#### 3.1.4. Mechanical Properties

From the mechanical point of view, CaP bioceramics are polycrystalline materials that brittle easily. Thus, their mechanical properties are influenced by crystallinity degree, grain size, grain boundaries, porosity, composition, presence of impurities, and manufacturing processes [141].

One of the weak points of the BCP is its compressive strength, which was reported in literature to be about three times lower than that of the human bone [142,143,144]. Sustained efforts were made to overcome the weakness of these materials, and various solutions were found to improve the mechanical properties. One of these solutions is to sinter BCP powders at high temperatures.

Bas et al. [98] evaluated the effects on the density, microhardness, and compressive strengths of salmon-derived CaPs sintered at different high temperatures (1000–1300 °C). They reported the raise of density values from 2.01 (1000 °C) to 2.96 g/cm^3^ (1300 °C). In case of microhardness behavior, a significant increase of the values in the temperature range of (1000–1100) °C was recorded. This characteristic was correlated to the change in porosity and density as an effect of particles’ coalescence. Values of Vickers microhardness measurements performed at 1000 and 1300 °C were 76 HV and 453 HV. The highest compressive strength value (116 MPa) and the maximum elastic modulus (633 MPa) were determined after sintering at 1100 °C in comparison to those obtained at 1200 and 1300 °C, probably because of phase dissociation with the temperature increase [98].

Another solution that proved to be efficient to improve mechanical properties was the addition of reinforcements, such as ceramics [95,145], metals, or polymers, which are well known as biocomposites or hybrid biomaterials [146]. However, there are reports on the improvement of polymers’ mechanical properties with the addition of CaP as powders or granulates in a polymer matrix [110,147,148,149].

Kiyochi Junior et al. [95] reported on the combination of niobium pentoxide (Nb_2_O_5_), with BCP as a promising nanocomposite with properties as similar as possible compared to those of human bones.

The density of the Nb-BCP nanocomposite was 17% greater than that of BCP (2.7 vs. 2.3 g/cm^3^). In comparison to HA powder obtained from sheep bones mixed with 5 and 10 wt.% niobium oxides, the Nb-BCP density was greater [150], probably because of the materials’ crystallinity, atmosphere and sintering time, grinding parameters, morphology, and particle size of the used precursor powders. The Nb-BCP at a 1:1 ratio resulted in an improvement of Vickers microhardness with 66% (0.78 HV) compared to that of pure BCP samples (0.47 HV). The compressive strength of the Nb-BCP sample was 95.4 MPa, which was much higher (up to 180%) than that of pure BCP (34.12) [95]. This value is comparable with the one for human bones, which varies from about 90 to 180 MPa depending on several factors: age, gender, kind of bone, and the compression direction [142,143,144].

#### 3.1.5. Biological Characteristics

Biocompatibility of a material is a complex process influenced by the following factors:

1. Interaction with the surroundings in terms of: allergic or toxicological effects, carcinogenic or mutagenic reactions, inflammatory processes, degree and quality of the biodegradation, and contact with human blood;
2. Best period of the implant application: long- or short-term periods;
3. Surface characteristics of the implants for the host tissue (chemical, biological, and morphological);
4. Structural biocompatibility between the mechanical properties of the implant and of the host tissue;
5. Function: optimal mechanical properties demanded by the application; and
6. Proportion: optimal implant size and shape.

On the other hand, the biocompatibility of a material strongly depends on the type of the application.

In vitro cytotoxicity evaluation of the powder obtained from three fish species revealed that the cell viability increased with the calcination temperature from 700 to 1100 °C. The cell viability of materials calcined at 1000 °C and 1100 °C was >100%, higher than that for 700 and 800 °C (i.e., 97–98% for *Salmo salar* powder), whilst the absorbance values of each sample had almost the same value. For all three powders, a cell viability >70% was demonstrated, which confirmed the noncytotoxic behavior of the materials, according to the International Standard ISO 10993-5:2009. After the co-culture of all the prepared material extracts and mesenchymal stem cells (MSCs) for 24 h, the in vitro cytotoxicity evaluation confirmed that the BCP materials produced from calcined fish bones had no cytotoxicity potential [93].

To evaluate the in vitro biocompatibility of the salmon-derived BCP powders using osteosarcoma (Saos-2) cells, Bas et al. [98] performed cytotoxicity tests with methylthiazol tetrazolium (MTT). These results indicated that BCP powders obtained at different calcination temperatures had no cytotoxic effect in comparison to control. Cell proliferation had the best result in the case of powder calcined at 1300 °C on the seventh day of the study [98].

The noncytotoxic effect was reported also by Boutinguiza et al. in their study [13] about BCP derived from fish discards. Based on their MTT tests, the cells produced large amounts of colored formazan, which indicated that all tested materials were non-cytotoxic. The same MTT assay evidenced the presence of nontoxic amounts of heavy metals in the BCP powders [13].

Piccirillo et al. [100] investigated the cytotoxicity and bioactivity of single-phase HA and biphasic HA/β-TCP derived from CaCl_2_ treated and untreated Atlantic cod fish bones in form of powder or pellets. To prove the material’s bioactivity, the pellets were immersed in SBF for different periods of time. At the surface of pellets fabricated from untreated bones, the formation of an apatite layer after only seven days was indicated. For the pellets obtained from CaCl_2_-treated bones, even after 28 days, there was still evidence of the partial covering of the pellets’ surface with small and irregular grains. These results indicate that the biphasic material (untreated bones) showed much higher bioactivity than the single-phase HA (CaCl_2_-treated bones). The cytotoxicity tests on Saos-2 cells showed that both materials were non-cytotoxic both in powder and in pellet form. One of the most important biological features of a material in contact with blood is considered to be its hemocompatibility. The tests were performed for both materials in contact with erythrocytes. The percentage of hemolysis was found to be less than 2%, which according to ASTM: F 756-00, means that all samples showed no hemolytic activity.

In vivo evaluation of BCP and Nb-BCP nanocomposite discs inserted in male adult Wistar rats (*Rattus norvegicus, var. albinus*) was reported by Kiyochi Junior et al. [95]. Both BCP and nanocomposite discs were shown to be biocompatible, bioactive, and osteoconductive.

### 3.2. BCP Coatings

#### 3.2.1. Morphological Characteristics

**Figure 8:**
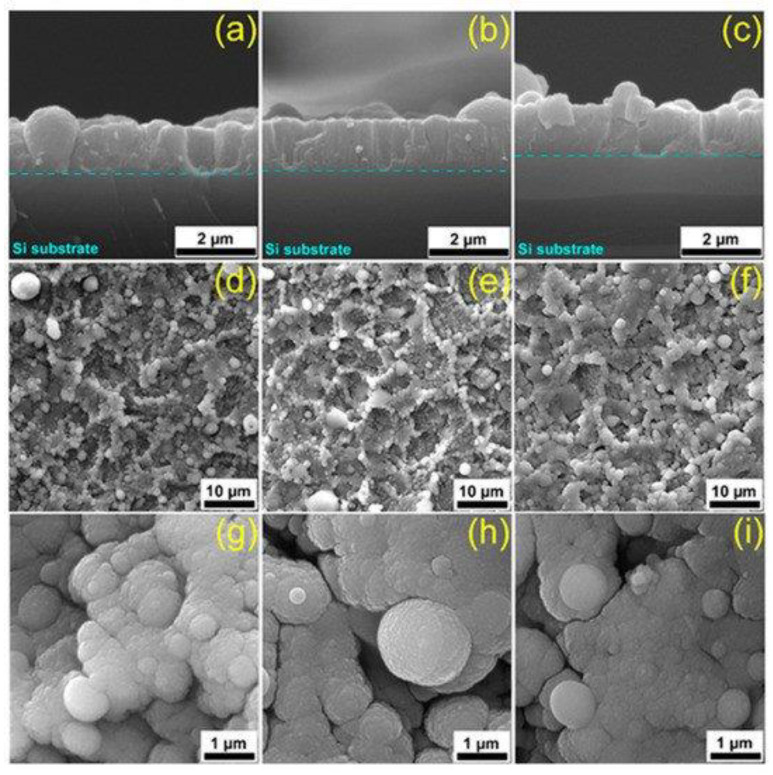
Cross-section (**a**–**c**) and top-view (**d**–**h**) SEM micrographs of (**a**,**d**,**g**) commercial HA, (**b**,**e**,**i**) Spa-BCP, and (**c**,**f**,**h**) Sal-BCP pulsed laser deposition (PLD) coatings, respectively. Reproduced with permission from [113]. Copyright MDPI, 2020.

Cross-sectional SEM images performed by Popescu-Pelin et al. [113] on the PLD-synthesized coatings showed an intimate film–substrate interaction. The BCP coatings’ average thickness was estimated to be ~1 µm. At lower magnification, the surface micrographies evidenced a rough morphology constituted of quasi-spheroidal particles (Figure 8), which are known to be characteristic to the PLD process [151,152].

When performing a comparison between the PLD coatings, similar topologies in the form of cauliflower-like aggregates could be observed. At higher magnification, these large structures were shown to be made up of grains with defined boundaries and diameters in the range of (0.09–2.5) µm.

**Figure 9:**
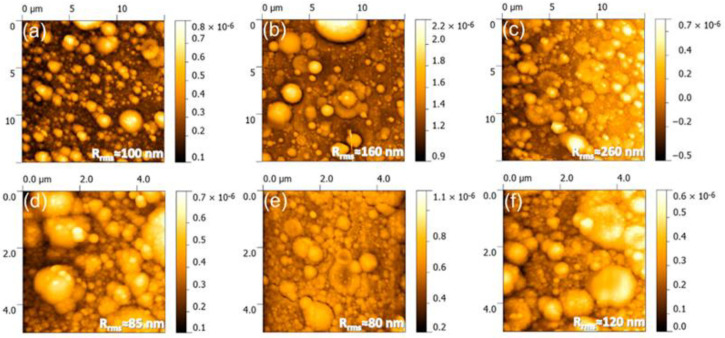
AFM images of rough surfaces (particles of 50–200 nm) of PLD coatings obtained from (**a**,**d**) commercial HA, (**b**,**e**) sea bream-BCP, and (**c**,**f**) salmon-BCP powders. The images were recorded on (**a**–**c**) 15 × 15 µm^2^ and (**d**–**f**) 5 × 5 µm^2^ areas. Reproduced with permission from [113]. Copyright MDPI, 2020.

In the case of AFM measurements, particles with dimensions in the (50–200) nm domain were revealed to spread between micro-aggregates (Figure 9). Their combination determined a rough surface of the PLD-synthesized coatings, with inferred roughness values higher than 80 nm and mainly influenced by the β-TCP content [113].

One should note here that these rough surfaces were demonstrated to positively influence osseointegration by stimulating protein adhesion and the differentiation of bone cells [153].

**Figure 10:**
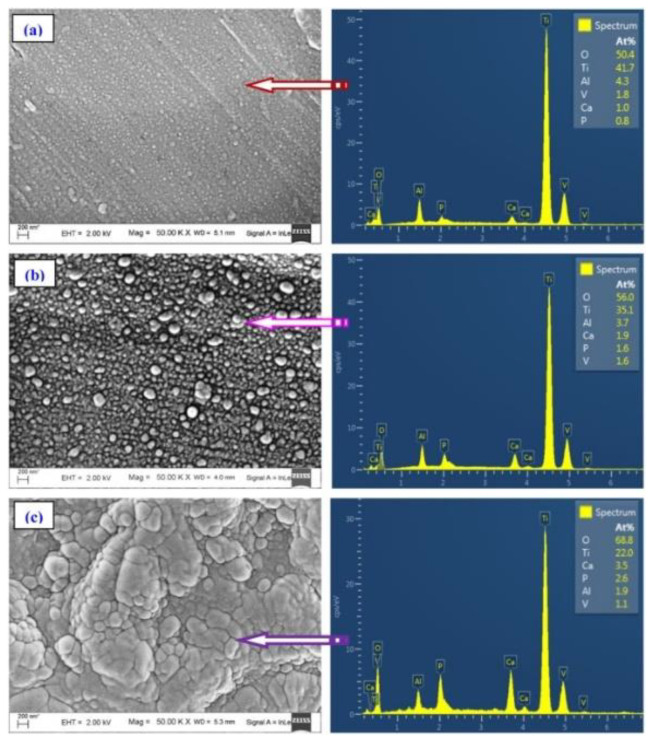
Surface morphology and corresponding EDS analysis of different thick films: (**a**) 400 nm, (**b**) 700 nm, and (**c**) 1000 nm. Reproduced with permission from [40]. Copyright Elsevier, 2018.

SEM micrographs corresponding to BCP coatings of raw fish scales origin deposited by rf-MS technique (at different thicknesses of 400, 700, and 1000 nm, respectively) [40] evidenced a dense and uniform morphology made up of regular grains, which were uniformly distributed onto the surface. The thinner film presented small and spherical grains, with average diameters of 48 nm. The 700-nm-thick BCP film evidenced bigger grains, with mean diameters of 121 nm. In the case of the thickest BCP film, bigger, elongated grains of 379 nm were observed (Figure 10).

Their dimensions were a consequence of the coalescence phenomenon [40]. The profilometric investigations revealed that the roughness value increased (from 94 to 153 nm) with the BCP films’ thickness. This observation was attributed both to the nucleation of spherical grains, which increases on rougher coated surfaces in comparison to uncoated ones, and to the presence of bigger grains and islands of grains, which coalescence on the surfaces of thicker BCP coatings [40]. After immersion of BCP coatings for 14 days in SBF, small, globular, and elliptical-like structures were observed on their surfaces. Moreover, in comparison to non-soaked samples, an important increase in wt.% apatite was indicated for all immersed coatings, with an emphasis on the thickest one (~87 wt.%).

Behera et al. [114] used the same renewable resources (i.e., raw fish scales) to fabricate sputtered BCP coatings onto both polished and micro-textured Ti-6Al-4V substrates. SEM investigations evidenced a smooth and uniform morphology for BCP coatings deposited on bare substrates, whilst in the case of the BCP-coated-textured ones, a rough and irregular morphology was observed. In the latter case, small patches of BCP material were emphasized, which can be mainly determined by the surface texturing. In comparison, the value of the surface roughness for the textured substrates increased from 0.98 to 1.84 µm. Further, after the deposition of BCP coatings, an increase of the roughness from 0.14 to 2.07 µm was registered. This behavior was due to the existence of nonuniform distribution of BCP patches on the surfaces of the textured substrates [114]. After soaking in SBF, the morphological features of BCP coatings consisted of spherical apatite aggregates, whose number was superior for the BCP-textured substrates in comparison to BCP-bare ones. This was a consequence of the higher nucleation sites on the surfaces of the textured substrates [114].

As a general remark, we conclude that morphologies rich in protuberances, which means also an enlarged active surface area, promote faster protein adhesion and cell proliferation.

#### 3.2.2. Elemental Composition

For both BCP targets and films, besides the majority of Ca, P, and O, which are the main constituents of the mineral bone [113], trace amounts of Na, Mg, Si, and/or S elements were also detected. One should note that the latter are characteristic natural dopants of the bone mineral [154]. In the case of BCP targets, Ca/P molar ratios of ≈1.55 and ≈1.65 were inferred. A possible explanation for this behavior was related either to the partial decomposition of HA into β-TCP or a calcium-deficient HA, which is characteristic to the bone mineral phase [155]. To the difference of the targets, for all BCP films, Ca/P molar ratios with lower values were determined.

EDS spectra of BCP films showed higher values of Ca, P, and O elements with the thickness increase, besides their homogenous dispersion onto the sputtered surfaces [40,114].

#### 3.2.3. Structural Characteristics

XRD analysis performed by Popescu-Pelin et al. [113] revealed the presence of a series of supplemental diffraction peaks, which corresponded to the β-TCP phase in the case of *Sparus aurata* (SA)-BCP and *Salmo salar* (SS)-BCP coatings, for both targets and films. The thickness of the BCP coatings in the micron range determined the appearance of some reflections of the Ti substrates. The results of Rietveld analysis indicated a higher β-TCP content (~50 wt.%) for SS targets in comparison to SA ones (~31 wt.%). A decrease of the β-TCP concentration was observed for both SS (~38 wt.%) and SA (~12 wt.%) coatings. Using a dedicated software (i.e., MAUD), the authors determined that the crystallites were either flattened in the *c* direction (SA target) or a mixture of elongated and flattened (SS target). The structural alterations observed during the coatings’ deposition were found to be responsible for the shift of the IR bands position in the case of targets and BCP films [113]. The orthophosphate units of HA and β-TCP phases were evidenced by the IR absorption bands at lower wavenumbers. The existence of the ν_2_ bending (~877 cm^−1^) and ν_3_ asymmetric stretching (~1462 and 1417 cm^−1^) modes of HA films indicated a B-type carbonatation. As compared to pristine material, this carbonatation was more pronounced for the fish-bone-derived BCP films due to the heat treatments performed in water vapors.

Behera et al. [40] demonstrated by XRD investigations that their sputtered coatings were biphasic. The values (in wt.%) corresponding to HA and β-TCP were calculated using Equation (1) [40] and are introduced in Table 2.

The crystallite sizes increased with the thickness of the deposited BCP films. This was related to the increase of the deposition time when the sputtered atoms reach the crystallized nuclei, which from the thermodynamical point of view, represent supportive sites for the arriving atoms [40]. The same group demonstrated that surface texturing had no influence on the crystallite sizes, the values being ~26 nm [114]. For a complementary approach, the authors used Raman technique for an in-depth investigation of the BCP sputtered coatings. The Raman shift at 612 cm^−1^ [138] corresponded to β-Ca_3_(PO_4_)_2_ due to both O-P-O and O-P bending and stretching modes of the orthophosphate group. Moreover, the appearance of the band at 962 cm^−1^ is an important signature of the existence of a HA phase. In general, for pure HA, this band has a sharp intensity, but in the current case, it was shown to have a very low intensity, which clearly confirmed (next to XRD results) the biphasic nature of the sputtered BCP coatings [40].

Moreover, the phase analysis of the sputtered BCPs immersed in SBF coatings evidenced the existence of HA peaks only. It is important to note that the intensity of the (200) peak increased with the thickness, which is indicative for a preferential growth of HA along this direction. It seems that the coatings’ thickness had an important influence over the HA growth in the SBF solution. The broad aspect of the peaks and their low intensity corresponded to a good crystallinity of the HA film, which increased for thicker BCP coatings. As a consequence, the calculated apparent crystallite sizes increased from 32 to 35 nm.

#### 3.2.4. Wettability Behavior

The wettability of CaP-based surfaces plays an important role in the formation of the apatite layer and for cell growth and is influenced by surface’s chemistry and morphology. There are studies that reported that materials covered with various CaP thin films are hydrophilic in nature [156,157].

A comparison of contact angle (CA) values measured for bare (uncoated) Ti-6Al-4V- and BCP-coated substrates with different thicknesses was reported [40,114]. The inferred value of the SBF CA for uncoated substrates was of 89.6°. In the case of BCP depositions, the CA decreased from ~74° to ~61°, with the increase of the coating’s thickness from 400 to 1000 nm, respectively. The improvement of the hydrophilic behavior can be explained by the existence of electro-negatively phosphate anions, which attract the electro-positive (H^+^) ones from the SBF molecule [158]. In the case of the substrates with micro-dimpled texturing, an increase in the hydrophilic behavior (77° to 71°) with the overlapping factors (0, 25, and 50%, respectively) was observed. One can conclude that the roughness of textured substrates increased in comparison to non-textured ones. As a consequence, an enhancement in hydrophilicity was observed. A significant shift in the hydrophilic behavior was indicated after the deposition of the BCP coatings. Thus, the surfaces of the textured substrates became super hydrophilic, with CA values dropping down to 17–21°. In the case of non-textured substrates, the deposition of BCP coatings determined the appearance of hydrophobic surfaces (CA of 95°). This behavior was due to the deposition of grains with sizes and roughness in the nanometer range, which inhibited the spreading of the SBF droplet [159].

#### 3.2.5. Mechanical Characteristics

The bonding strength at the BCP film–substrate interface is an important characteristic for the fabrication of high-quality coatings with long-term in-situ stability.

The results of the pull-off tests performed by Popescu-Pelin et al. [113] showed different performance of the synthesized BCP coatings. Thus, SS coatings, with a higher β-TCP content, were easily detached from the metallic substrates, and the inferred adherence values were of (25–33) MPa. In the case of SA coatings with a lower β-TCP content, the inferred bonding strength values were in the (47–49) MPa range. One could therefore conclude that the increase of the β-TCP concentration in the deposited BCP coatings consequently determined a reduction of the adherence values. It is known that a minimum value of 15 MPa for the bonding strength adherence is imposed by the ISO-137792 Part 2 “Coatings of Hydroxyapatite” standard in the case of coatings considered for medical applications. In this respect, it is important to emphasize upon that all tested BCP coatings demonstrated superior values to this minimum threshold.

Behera et al. [40] reported a micro-hardness value of 324 HV for uncoated Ti-6Al-4V substrates. After the sputtering process of BCP coatings, the microhardness values increased significantly with the thickness and reached (430–455) HV. This result can be explained by the presence of almost the same HA and β-TCP content on the surface of the synthesized coatings. One could conclude that the thicker BCP coatings presented a slightly higher resistance to indent and thus an increased stability in comparison to thinner ones. Moreover, no delamination of the sputtered coatings was found, which was related to their good bonding strength adherence to the metallic substrates [40].

#### 3.2.6. Biological Characteristics

Solubility of SS and SA coatings in Dulbecco’s Modified Eagle medium (DMEM) supplemented with 10% fetal bovine serum (FBS) solution was evaluated by Popescu-Pelin et al. [113]. By immersion in proper medium for 24 h, a larger mass loss for SS coatings (7.5%) in comparison to SA ones (4%) was detected, mainly due to a higher β-TCP concentration in the first case. After three days of soaking, a considerably lower mass loss (3%) and a mass gain (6.4%) was indicated for the SS and SA coatings, respectively. It was thus suggested that the CaP precipitation could appear in homeostatic conditions, especially in the case of the SA coating (with a higher content of HA, which was demonstrated to elicit improved biomineralization capacity [160]). The modified morphology consisted of a thin layer of fine, acicular crystals, which uniformly covered the characteristic PLD particulates from the surface of the BCP coatings. This process was previously reported to indicate the CaP appearance in simulated body medium [161]. After seven days of immersion, the aforementioned trend was strengthened, and an even thicker CaP layer was formed, which totally modified the overall morphology of coatings [162].

The apatite layer formation was investigated by Behera et al. [40] by immersion of the BCP coatings in SBF solution. After 14 days of soaking, the β-TCP content decreased in comparison to non-immersed sputtered ones. Before soaking in SBF, the sputtered BCP coatings presented a HA content of ~44 wt.%. After immersion, it increased up to (68–87)% depending on the thickness of the sputtered coatings. This was due to an improvement in the nucleation sites with the deposition of a higher quantity of apatite.

Compared to bare substrates, an increased mass of adsorbed proteins was indicated by Behera et al. [114] for laser-textured surfaces (2.8 vs. 5.3 mg/cm^2^) due to the existence of protein-binding groups (i.e., Ca^2+^, PO_4_^3−^, OH^−^) in the BCP coatings. When protein adsorption was evaluated as a function of surface wetting behavior, one could observe that it increased with hydrophobicity. The protein mass adsorption on the hydrophilic BCP coatings deposited on laser-textured substrates was inferior to the one observed in the case of BCP-coated bare ones. After the adsorption process, the hydrophobic behavior of BCP-coated bare substrates became super-hydrophilic (CA ~ 29°), whilst the CA of BCP-textured surfaces increased from 17–21° to 50–80°, which indicated the protein layer formation. The cellular response of modified surfaces was also investigated using osteoblast cells (MG63). The higher number of spread MG63 cells was present on the BCP-coated textured surfaces. On BCP-coated bare substrates, cells with a round morphology and polygonal-shaped ones were observed, whilst for BCP-coated textured substrates, flattened cells with high density, uniform distribution onto the surface, and strong adherence were seen. The protein acts as a cushion for the adhering cells, which results in a more effective spreading and adherence of the osteoblasts [163]. The BCP-textured surfaces presented larger actin filaments than in the case of BCP-bare ones, which was indicative for their high cell adhesion and growth capability. It was concluded that protein adsorption, wettability, and surface roughness can simultaneously improve cells’ adhesion and spreading. The laser texturing of substrates improved proliferation rate and indicated a better cytocompatibility, which is suitable for bone regeneration. This behavior was due to the existence of both β-TCP and HA phases, which consequently led to higher protein adsorption, wettability, and surface roughness. In addition, the existence of large nuclei determined a higher cell-division rate, which resulted in an improved cell proliferation [114]. β-TCP phase has a higher solubility rate, which further determines a supersaturation of the SBF solution with Ca^2+^ and (PO_4_)^3−^ ions.

##### Cytocompatibility of Coatings

Using the fluorescence microscopy, similar morphological features of cells grown onto SA and SS coatings and control was shown. The grown cells presented numerous actin filaments, filopodia, and lamellipodia, which facilitate the contacts between the neighboring cells and their spreading. Using cytoplasmic lactate dehydrogenase (LDH) release in the media, no changes were observed after 24 h, which confirmed that the PLD synthesized coatings had no cytotoxic behavior [113].

##### Antibacterial Activity

The antibacterial activity on *Escherichia coli* was monitored after different incubation times (i.e., 24, 48, and 72 h, respectively), to investigate the inhibition capacity of the synthesized BCP coatings both in the case of the initial stages of microbial growth and biofilm development. A significant increase of the anti-biofilm performances was demonstrated after 24, 48, and 72 h of incubation (in the case of SA coatings) and 48 and 72 h only (in the case of SS ones) when compared to control samples. The BCP materials with a high content of β-TCP can provide a prolonged protection at 72 h against bacterial colonization due to the release of some trace elements, such as Na, Mg, Si, and S, some of which possess already established antimicrobial effects against *Pseudomonas aeruginosa*, *Staphylococcus aureus*, *Candida albicans,* and/or *E. coli* microbial strains [164,165,166].

### 3.3. BCP Scaffolds

The major role of the scaffolds is to balance temporary mechanical functions with mass transport of active substances to support biological delivery and tissue regeneration. Like in the case of any bone substitute, two important requirements for the production of scaffolds are: (i) to be fabricated of biocompatible materials (to never cause adverse immune responses in the host tissue after grafting) and (ii) a non-cytotoxic behavior of the degradation by-products.

#### 3.3.1. Morphological Characteristics

**Figure 11:**
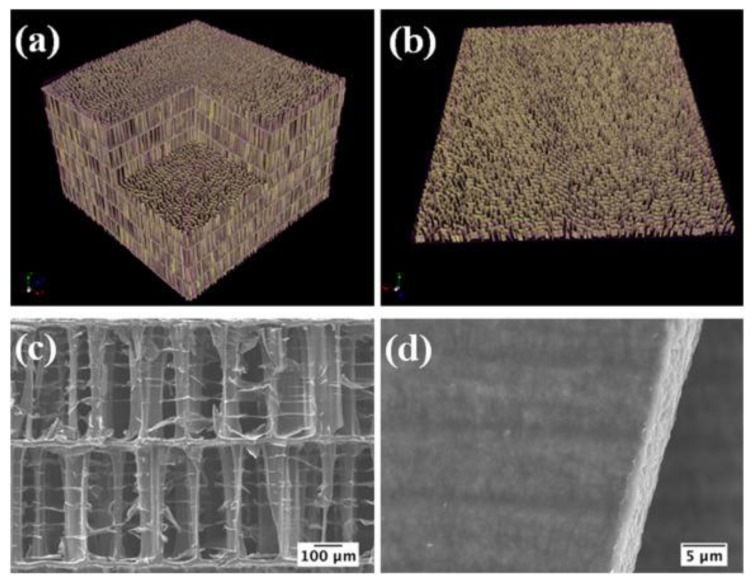
Microstructure of raw CB. (**a**,**b**) μ-CT images highlighting the interconnectivity and sigmoidal path of the pillars; SEM micrographs (**c**) showing the presence of β-chitin and (**d**) the detail of the pillar wall. Reproduced with permission from [106]. Copyright Elsevier, 2019.

SEM investigations showed a complex architecture of the cuttlefish bone scaffolds, which were made up of numerous pillars, and generated chambers with a height of ~400 μm and a wall thickness of ~10 μm. SEM results were confirmed by µ-CT investigations, which revealed the complex porosity of raw CB with interconnected layers (Figure 11) [106,119].

One should note that, after the heat treatment and sintering process, this porous morphology was maintained for all structures. In addition, the scaffolds’ porosity was not influenced by the thermal treatment. The determined values of 92–93% were similar to those obtained in the case of raw CB (92.8%). A slight decrease in porosity was registered for the polymeric coatings. Moreover, under higher magnification, the formation of CaP crystals was observed, and the morphological characteristics were not modified by the doping agents. It was also evidenced that the scaffolds’ surfaces were uniformly coated by the polymers, which presented various interactions with the inorganic matrix.

One should emphasize that the beneficial role of these particular structural characteristics of scaffolds for bone development was reported [167].

After 14 days of immersion in SBF, SEM investigation of the scaffolds evidenced morphologies composed of isolated or aggregated microspheres, which were demonstrated to be characteristic for the in vitro development of apatites [106].

In the case of BG coatings, the sintering process promoted the grains’ development. Moreover, the BG coating was demonstrated to have no negative effect on the pores. The surface morphology of the BG coatings evidenced the appearance of cracks, which generally appear in the case of structures fabricated by sol-gel method, during the application of drying and calcination processes. As a direct consequence, the value of the inferred BG coatings’ thermal expansion coefficient was almost four times smaller than the one corresponding to the BCP scaffolds (i.e., 2.3 vs. 8.2 × 10^−6^ C^−1^). After soaking in SBF for 14 days, the roughness values corresponding to the coated scaffolds were superior to the uncoated ones [119].

#### 3.3.2. Elemental Composition

EDS investigations revealed the presence of Ca, P, and O elements along with trace amounts of Na, Mg, and Cl, which are generally met in the composition of healthy human bone [106]. Further, the successful incorporation of Sr^2+^, Mg, and Zn doping agents was evidenced [119].

#### 3.3.3. Structural Characteristics

The XRD investigations of undoped and doped BCP scaffolds sintered at 1200 °C evidenced the existence of both HA and β-TCP phases, with proportions dependent on the nature of the dopants. Thus, when using Sr^2+^ only, all peaks were shifted to lower angles. In the case of a mixture between Sr^2+^ and Mg^2+^ or Zn^2+^, the XRD patterns showed an increase of the intensity along with a shift to higher angles of the peaks corresponding to the β-TCP phase, whilst the HA peaks were shifted in the opposite direction [106].

In a complementary study, the BG films fabricated by sol-gel method were shown to have an amorphous character. One should note that similar patterns (for HA and β-TCP) in the case of scaffolds before and after the impregnation with BG films (calcined at 700 °C, 2 h) were evidenced. Before the BG coating, the relative percentages of the HA and β-TCP crystalline phases were of 63.9% and 36.1% in comparison to 64.6% and 35.4%, estimated after the BG coating [119].

In both studies reported by Neto et al. [106,119], FT-IR results demonstrated the characteristic vibrational modes of –PO_4_ and –OH groups. The results of the thermal analysis (DTA and TG) of raw cuttlefish bones revealed a weight loss of 1.02% up to 150 °C, associated with water evaporation. The weight loss between 150 °C and 500 °C due to the burning of the organic matter was about 4.49%. Finally, the weight loss of 38.64% over (500–1000) °C was due to the decomposition of aragonite into calcium oxide [106].

#### 3.3.4. Mechanical Properties

After the heat treatment and sintering processes, the compressive strength of the raw CB was reduced by ~8 times in the case of BCP, BCP-Sr, and BCP-SrZn structures (1.6 vs. 0.2 MPa) and by ~4 times for BCP-SrMg and BCP-SrMgZn, whilst the polymeric coatings were shown to significantly increase the value of the compressive strength [106].

Neto et al. evidenced that the characteristic stress-strain curves of the uncoated scaffolds underwent a layer-by-layer gradual collapse, whilst in the case of the coated ones, this behavior was diminished. Moreover, it was demonstrated that the role of the BG coating was to improve the overall mechanical performances of the BCP scaffolds [119].

#### 3.3.5. Biological Characteristics

Owing to their ability to form a bone-like HA layer, BG materials were demonstrated to bond firmly to living tissues [168,169]. Thus, the BG coatings were shown to improve the in vitro bioactivity of the scaffolds.

### 3.4. BCP Nanorods

The BCP nanorods obtained from cuttlefish bones were characterized from the structural and morphological points of view. XRD pattern confirmed the formation of a biphasic mixture based on the presence of HA and β-TCP corresponding peaks [99]. The BCP morphology consisted of nanorods with ~230 nm in diameter and (1–1.2) μm length. To improve the mechanical and biological properties of polyvinyl alcohol (PVA) hydrogels, BCP nanorods were used as a reinforcement material and combined with this polymer in different concentrations, from 1.5 to 3.5 wt.%, respectively. The obtained PVA hydrogels had a porous structure with an average pore size of 12.3 μm. The pore dimensions showed a decreasing tendency with the increase of BCP reinforcement concentration [99]. In general, the surface porosity promotes mechanical interlocking between the sample and surrounding tissues, which could lead to an improvement of the mechanical stability of an implant [170]. The existence of the two components in BCP and of the PVA in the hydrogel composition were also confirmed by FTIR investigations, where the characteristic peaks for phosphate ions (1200–550 cm^−1^) [171] and crystalline PVA (1098 cm^−1^) [172] were evidenced. From the mechanical point of view, the reinforcement with BCP had the best results following tensile strength evaluation of PVA mixed with 2.5 wt.% BCP. The value of tensile strength increased from 0.41 MPa (for PVA only) with a significant deformation of 281%, up to 5.2 MPa, with 363% deformation. The tensile modulus increased from 0.5 kPa to 613 kPa and exhibited an enhancement in the stiffness of the hydrogel. For the compressive strength, the best result was 14.9 MPa, with a deformation of 72% [99].

The biological evaluation of the PVA with 2.5 wt.% BCP hydrogel revealed that this combination had positive effects as antimicrobial materials. Thus, the inhibition of gram-negative *E. coli*, gram-positive *S. aureus,* and *C. albicans* fungi by 28%, 20% and 26%, respectively, after 12 h of culture [173] was demonstrated. The combination with 2.5 wt.% BCP increased cell proliferation from 82% to 93%. This was due to the release of bioactive Ca^2+^ and (PO_4_)^3−^ ions from HA and TCP phases, which help surrounding tissue to promote cell adhesion and proliferation [174]. The composite hydrogels proved to be biocompatible with over 80% of cell-viability response of fibroblast cells.

### 3.5. Applications of BCP Materials

Fish discards can be used as an alternative adsorbent biomaterial due to their wide availability. Over the years, scientific reports demonstrated that fish discards may be important sources of natural HA, which were highly valuated in many domains, including the industry of manufacturing functional foods, as drug delivery agents, biomaterials, and various biomedical by-products [91,175].

The domain in which both synthetic and natural BCP materials found their general application is the bone-tissue engineering [176]. Because CaP materials are brittle, and their fatigue resistance is quite limited, BCPs were successfully used as bone-defect fillers [177]. Moreover, the osseointegration of dental and orthopedic implants were improved by covering implants with BCP materials by means of various deposition techniques [114,178]. Additionally, for soft tissue replacement, BCP nanorods were used for reinforcement of PVA hydrogels [99].

In the pharmaceutical field, BCP was used in manufacturing complex formula for the treatment and/or prevention of osteoporosis. For the oral hygiene, BCP was utilized as a soft polish for tooth enamel, as a tooth re-mineralizing agent, and an additive for toothpaste.

Another application of BCP powders could be as a precursor material for the production of nanoparticles [179], which can be incorporated into sunscreen creams [180].

Based on their capability to exchange ions, apatite minerals can be applied for heavy-metal removal during the treatment of waste waters and soils. The principle is that the calcium ion is replaced by bivalent heavy-metal ions [181,182].

Besides their biological activities explored in the pharmaceutic, nutrition, and cosmetic industries [183], BCP-derived materials can contribute to the increase of the added-value in food products, helping fats’ binding, solubility, and water-holding capacity and acting as emulsifying and foaming agents [184,185]. In addition, they can act as an additive in multivitamin complexes and as texturizing and anti-caking agents in flours, milk powder, lyophilized products, and soup powders. Last but not least, BCP materials can be considered a slow-acting fertilizer for acid soils. The different degree of water-solubility of the two major phases of BCP (β-TCP greater than HA) causes phosphorus to be released in a sustained manner over time [90].

CaP particles from *Salmo salar* bones in combination with humic substances were recently reported by Adamiano et al. [186] as biostimulants for the growth of *Diplotaxis tenuifolia* and *Valerianella locusta*.

## 4. Conclusions

This review summarizes the results published in papers related to BCP materials starting from marine resources (i.e., fish discards only). Large quantities of fish discards (i.e., heads, bones, skins, and viscera) are generated by the fish processing industries, and there is a constant need for efficient ways to transform these wastes into valuable biomaterials.

The advantages of choosing fish discards are numerous. They are low-cost and abundant materials that can be easily transformed into resources of calcium phosphates (CaP). Reports from the literature demonstrated that the overall characteristics of BCP materials obtained from fish discards are superior to single-phase CaP ones (i.e., hydroxyapatite, HA or beta-tricalcium phosphate, β-TCP). It is known that HA has a poor biodegradation rate, whilst β-TCP is characterized by high chemical stability, good biological activity, and favorable biodegradation rate.

BCP materials present osteoconductive potential without any growth factors, which recommends them for bone substitution. Additionally, the adsorption of protein is enhanced in the case of BCP due to hydrophobic interactions, resulting in an improved osteoconductivity as compared to HA.

Another advantage of BCPs is that they are versatile materials that can be utilized in various forms, such as powders, coatings, nanorods, or scaffolds. In addition, they can be combined with other materials, for example, ceramics, polymers, or metals, to achieve novel and enhanced properties in dedicated applications.

One of the most applied methods to synthesize fish discards is calcination. BCP materials can be obtained at high temperatures, usually over 900 °C, and their properties strongly depend on the calcination temperature. With increasing of calcination temperature, an increase of particles sizes along with a decrease of the surface specific area were observed. The porous-shaped structure of BCP powder has the advantage of leading to a fast osteointegration.

Despite these advantages, the mechanical properties of BCP materials are weak and need to be improved, especially for biomedical applications. There is a limitation of BCP materials related to their compressive strength, which is much lower than the one of human bone. One possible solution to tackle this drawback is to sinter the BCP powders at high temperatures. Other weak points are the small values for microhardness and elastic modulus, which can be overcome by combining BCP materials with other biocomposites, such as ceramics, metals, or polymers.

Furthermore, the composition and structural characteristics of the different sample architecture of BCP materials can induce an appropriate cell proliferation, which is a key-process for a fast bone-tissue regeneration. BCP materials present non-stoichiometric values of Ca/P molar ratio. The values inferior to 1.67 could be attributed to a partial transformation of the HA phase in β-TCP and, consequently, to the formation of BCP compound, whilst those superior to 1.67 are due to the presence of carbonate ions, which substituted the phosphate ones. Besides the major Ca and P elements, BCP materials contain also trace mineral elements (i.e., Mg, Na, K, and Sr), which are known to enhance osteogenesis when used as bone substitutes. Narrow and well-defined peaks of BCP powder materials with an average crystallite size resembling the one of the mature bones (i.e., ~50 nm) were found to be dependent on the calcination temperature.

In conclusion, BCP materials derived from fish discards were shown to have a huge potential and impact not only in the biomedical field but also in other important domains (i.e., cosmetics, food industry, and agriculture). In our opinion, this review could be considered as a starting point towards future applications of BCP materials for the improvement of human lives and for reaching zero-waste management of fish discards.

## Figures and Tables

**Table 1:** Preparation of BCP materials derived from various fish discards.

Fish Discards	Prepared Form	Pre-Treatment	Calcination		Characterization Techniques	Ref.
			[°C]	Time [min]		
Red scorpionfish (*Scorpaena scrofa*), Atlantic horse mackerel (*Trachurus trachurus*), salmon (*Salmo salar*) bones	Powders	Alkaline hydrolysis	750, 900		FEG/HR-SEM, EDS, XRD, FTIR	[109]
Black scabbardfish (*Aphanopus carbo*) bones, grey triggerfish (*Balistes capriscus*) skin	Powders	Washed in hot water	900, 950, 1000, 1100, 1200	-	FAAS, XRD, FT-IR	[85]
	Powders	Washed in hot water and dried at 60 °C	800, 1000	60	XRD, Raman	[96]
Sea bream (*Sparus aurata*), salmon (*Salmo salar*) bones	Powders and thin films	NaOH solution	850	-	SEM, EDS, XRD, FT-IR, bonding strength, solubility/bioactivity, cytocompatibility, antibacterial activity tests	[113]
Black scabbardfish (*Aphanopus carbo*) bones, grey triggerfish (*Balistes capriscus*) skin	Powders	-	400, 600, 800, 1000	-	XRD, FT-IR, ICP-OES, SEM/FEG-SEM, SSA	[121]
Fish otoliths	Powders	Washed with ethanol and deionized water for 1 h	1000	-	XRD, FT-IR, Raman, SEM	[92,122]
Native Brazilian fish (*Pseudoplatystoma* *corruscans*) bones	Powders/discs	Cleaned with hot water, washed	900		XRD, Microhardness, Optical microscopy,	[95]
Cuttlefish (*Sepia officinalis*) bones	Polymeric coating of scaffolds	Washed with water, dried	-	-	XRD, ICP-OES, FT-IR, SEM, µ-CT, compression tests, SBF	[106]
	BG coating of scaffolds	-	700	120	XRD, FT-IR, SEM, µ-CT, compression tests, SBF	[119]
	Nanorods	Dipped in boiling water for 1 h	900	240	SEM, FT-IR, porosity, CA, mechanical, tribological tests, antimicrobial, cytotoxicity	[99]
*Salmo salar*, *Anoplopoma fimbria*, sardine bones	Powder	Boiled in deionized water for 2 h, washed with flowing water	600–1100	60	XRD, FT-IR, SEM, XRF, cell culture, cytotoxicity	[93]
Sardine scales	Powder	Washed with hot and distilled water	1000	–	XRD, IR	[97]
Salmon (*Salmo salar)* bones	Powder/cylindrical pellets	Boiled in water for 1 h, treated with 1% NaOH solution, washed with ultra-pure water	800	180	XRD, SEM, EDS, FT-IR, microhardness, in vitro biocompatibility tests	[98]
	Powder	Boiled in deionized water for 2 h, washed with flowing water	600	60	XRD, FT-IR, SEM, Cytotoxicity, Western blotting analysis	[94]
Fish (*Tilapia nilotica*) scales	Powder/films	Washed in distilled water, dried	800	–	XRD, SEM, Raman, profilometry, contact angle, mechano-tribological tests, in vitro bioactivity	[40]
	Powder	Washed in distilled water	–	–	TEM, XRD, FT-IR, TGA	[91]
Sword fish (*Xiphias gladius*), tuna (*Thunnus thynnus*)	Powder	Boiled in water for 1 h, washed in strong water jet	600, 950	720	FE-SEM, TEM/HRTEM, EDX, XRD, FT-IR, ICP-OES, Raman	[13]
Hairtails fish bones	Powder	Boiled in water for 3 h	700, 800, 900	60	XRD, FT-IR, XRF, SEM	[32]
Pintado (*Pseudoplatystoma corruscans*) bones	Powder		900	480	XRD, SEM, EDS hardness tests	[105]
Pintado (*Pseudoplatystoma corruscans*), jaú (*Paulicea lutkeni*), cachara (*Pseudoplatystoma fasciatum*) bones	Powder		900	480	XRD, SEM, FT-IR	[110]

**Table 2:** HA and β-TCP phase content (wt.%) and crystallite sizes of BCP coatings with various thickness.

BCP Coating Thickness (nm)	HA (wt.%)	β-TCP (wt.%)	Crystallite Sizes (nm)
400	43.4	56.6	15
700	43.5	56.5	17
1000	44.9	55.1	18

## References

[1] Alliedmarketresearch. https://www.alliedmarketresearch.com/implantable-medical-devices-market.

[2] Oladele I.O., Agbabiaka O.G., Olasunkanmi O.G., Balogun A.O., Popoola M.O. (2018). Non-synthetic sources for the development of hydroxyapatite. J. Appl. Biotechnol. Bioeng..

[3] Akram M., Ahmed R., Shakir I., Ibrahim W.A.W., Hussain R. (2014). Extracting hydroxyapatite and its precursors from natural resources. J. Mater. Sci..

[4] Tite T., Popa A.-C., Balescu L.M., Bogdan I.M., Pasuk I., Ferreira J.M.F., Stan G.E. (2018). Cationic Substitutions in Hydroxyapatite: Current Status of the Derived Biofunctional Effects and Their In Vitro Interrogation Methods. Materials.

[5] Abidi S.S.A., Murtaza Q. (2014). Synthesis and characterization of nanohydroxyapatite powder using wet chemical precipitation reaction. J. Mater. Sci. Technol..

[6] Bose S., Tarafder S., Bandyopadhyay A., Mucalo M. (2015). Hydroxyapatite coatings for metallic implants. Hydroxyapatite (HAp) for Biomedical Applications.

[7] Kang Z., Niu J.Z.L. (2018). A one-step hydrothermal process to fabricate superhydrophobic hydroxyapatite coatings and determination of their properties. Surf. Coat. Tech..

[8] Ciobanu C.S., Popa C.L., Predoi D. (2016). Cerium-doped hydroxyapatite nanoparticles synthesized by the co-precipitation method. J. Serb. Chem. Soc..

[9] Raucci M.G., Demitri C., Soriente A., Fasolino I., Sannino A., Ambrosio L. (2018). Gelatin/nano-hydroxyapatite hydrogel scaffold prepared by sol-gel technology as filler to repair bone defects. J. Biomed. Mater. Res. Part A.

[10] Duta L., Mihailescu N., Popescu A.C., Luculescu C.R., Mihailescu I.N., Çetin G., Gunduz O., Oktar F.N., Popa A.C., Kuncser A. (2017). Comparative physical, chemical and biological assessment of simple and titanium-doped ovine dentine-derived hydroxyapatite coatings fabricated by pulsed laser deposition. Appl. Surf. Sci..

[11] Oktar F.N. (2007). Microstructure and mechanical properties of sintered enamel hydroxyapatite. Ceram. Int..

[12] Rey C., Combes C., Drouet C., Glimcher M.J. (2009). Bone mineral: Update on chemical composition and structure. Osteoporos. Int..

[13] Boutinguiza M., Pou J., Comesaña R., Lusquiños F., De Carlos A., León B. (2012). Biological hydroxyapatite obtained from fish bones. Mater. Sci. Eng. C-Mater. Biol. Appl..

[14] Venkatesan J., Lowe B., Manivasagan P., Kang K.-H., Chalisserry E.P., Anil S., Kim D.G., Kim S.-K. (2015). Isolation and Characterization of Nano-Hydroxyapatite from Salmon Fish Bone. Materials.

[15] Coelho T.M., Nogueira E.S., Weinand W.R., Lima W.M., Steimacher A., Medina A.N., Baesso M.L., Bento A.C. (2007). Thermal properties of natural nanostructured hydroxyapatite extracted from fish bone waste. J. Appl. Phys..

[16] Iriarte-Velasco U., Sierra I., Zudaire L., Ayastuy J.L. (2015). Conversion of waste animal bones into porous hydroxyapatite by alkaline treatment: Effect of the impregnation ratio and investigation of the activation mechanism. J. Mater. Sci..

[17] Trinkunaite-Felsen J., Birkedal H., Zarkov A., Tautkus S., Stankeviciute Z., Kareiva A. (2016). Environmentally benign fabrication of calcium hydroxyapatite using seashells collected in Baltic Sea countries: A comparative study. Silicon Relat. Elem..

[18] Terzioğlu P., Öğüt H., Kalemtaş A. (2018). Natural calcium phosphates from fish bones and their potential biomedical applications. Mater. Sci. Eng. C.

[19] Herliansyah M.K., Hamdi M., Ide-Ektessabi A., Wildan M.W., Toque J.A. (2009). The influence of sintering temperature on the properties of compacted bovine hydroxyapatite. Mater. Sci. Eng. C.

[20] Iafisco M., Ruffini A., Adamiano A., Sprio S., Tampieri A. (2014). Biomimetic magnesium–carbonate-apatite nanocrystals endowed with strontium ions as anti-osteoporotic trigger. Mater. Sci. Eng. C.

[21] Chandran S., Shenoy S.J., Babu S.S., Nair R.P., Varma H.K., John A. (2018). Strontium Hydroxyapatite scaffolds engineered with stem cells aid osteointegration and osteogenesis in osteoporotic sheep model. Colloids Surf. B Biointerfaces.

[22] Pietak A.M., Reid J.W., Stott M.J., Sayer M. (2007). Silicon substitution in the calcium phosphate bioceramics. Biomaterials.

[23] Leventouri T.H. (2006). Synthetic and biological hydroxyapatites: Crystal structure questions. Biomaterials.

[24] Ideia P., Pinto J., Ferreira R., Figueiredo L., Spínola V., Castilho P.C. (2020). Fish processing industry residues: A review of valuable products extraction and characterization methods. Waste Biomass Valor..

[25] Best S.M., Porter A.E., Thian E.S., Huang J. (2008). Bioceramics: Past, present and for the future. J. Eur. Ceram. Soc..

[26] Hoyer B., Bernhardt A., Heinemann S., Stachel I., Meyer M., Gelinsky M. (2012). Biomimetically Mineralized Salmon Collagen Scaffolds for Application in Bone Tissue Engineering. Biomacromolecules.

[27] Rincón-López J.A., Hermann-Muñoz J.A., Giraldo-Betancur A.L., De Vizcaya-Ruiz A., Alvarado-Orozco J.M., Muñoz-Saldaña J. (2018). Synthesis, Characterization and In Vitro Study of Synthetic and Bovine-Derived Hydroxyapatite Ceramics: A Comparison. Materials.

[28] Eliaz N., Metoki N. (2017). Calcium Phosphate Bioceramics: A Review of Their History, Structure, Properties, Coating Technologies and Biomedical Applications. Materials.

[29] Miculescu F., Stan G.E., Ciocan L.T., Miculescu M., Berbecaru A., Antoniac I. (2012). Cortical bone as resource for producing biomimetic materials for clinical use. Dig. J. Nanomater. Biostruct..

[30] Niakan A., Singhbe R., Ganesan P., Tan C., Purbolaksono J., Chandran H., Teng W. (2015). Sintering behaviour of natural porous hydroxyapatite derived from bovine bone. Ceram. Int..

[31] Ramirez-Gutierrez C.F., Palechor-Ocampo A.F., Londoño-Restrepo S.M., Millán-Malo B.M., Rodriguez-García M.E. (2015). Cooling rate effects on thermal, structural, and microstructural properties of bio-hydroxyapatite obtained from bovine bone. J. Biomed. Mater. Res. Part B Appl. Biomater..

[32] Zhang L., Zhang C., Zhang R., Jiang D., Zhu Q., Wang S. (2019). Extraction and characterization of HA/b-TCP biphasic calcium phosphate from marine fish. Mater. Lett..

[33] Nery E., Lynch K., Hirthe W., Mueller K. (1975). Bioceramic implants in surgically produced infrabony defects. J. Periodontol..

[34] LeGeros R.Z. (1988). Calcium phosphate materials in restorative dentistry: A review. Adv. Dent. Res..

[35] Moore D., Chapman M., Manske D. (1985). Evaluation of a new biphasic calcium phosphate ceramic for use in grafting long bone diaphyseal defects. Trans. Annu. Meet. Soc. Biomater. Conjunction Interna.

[36] Ellinger R.F., Nery E.B., Lynch K.L. (1986). Histological assessment of periodontal osseous defects following implantation of hydroxyapatite and biphasic calcium phosphate ceramics: A case report. Int. J. Periodontics Restor. Dent..

[37] LeGeros R.Z. (1986). Variability of HAP/β-TCP ratios in sintered apatites. J. Dent. Res..

[38] Daculsi G., Legeros R.Z., Nery E., Lynch K., Kerebel B. (1989). Transformation of biphasic calcium phosphate ceramics in vivo: Ultrastructural and physicochemical characterization. J. Biomed. Mater. Res..

[39] Suneelkumar C., Datta K., Srinivasan M.R., Kumar S.T. (2008). Biphasic calcium phosphate in periapical surgery. J. Conserv. Dent..

[40] Behera R.R., Das A., Pamu D., Pandey L.M., Sankar M.R. (2018). Mechano-tribological properties and in vitro bioactivity of biphasic calcium phosphate coating on Ti-6Al-4V. J. Mech. Behav. Biomed. Mater..

[41] Kim T.-W., Lee H.-S., Kim D.-H., Jin H.-H., Hwang K.-H., Lee J.K., Park H.-C., Yoon S.-Y. (2012). In Situ Synthesis of Magnesium-Substituted Biphasic Calcium Phosphate and in Vitro Biodegradation. Mater. Res. Bull..

[42] Samavedi S., Whittington A.R., Goldstein A.S. (2013). Calcium Phosphate Ceramics in Bone Tissue Engineering: A Review of Properties and Their Influence on Cell Behavior. Acta Biomater..

[43] Bouler J.M., Pilet P., Gauthier O., Verron E. (2017). Biphasic calcium phosphate ceramics for bone reconstruction: A review of biological response. Acta Biomater..

[44] Goyenvalle E., Aguado E., Nguyen J.M., Passuit N., Le Guenennec L., Layrolle P., Daculsi G. (2006). Osteointegration of femoral stem prostheses with a bilayered calcium phosphate coating. Biomaterials.

[45] Shim K., Kim S., Yun Y., Jeon D., Kim H., Park K., Song H. (2017). Surface Immobilization of biphasic calcium phosphate nanoparticles on 3D printed poly(caprolactone) scaffolds enhances osteogenesis and bone tissue regeneration. J. Ind. Eng. Chem..

[46] Marques C., Olhero S., Abrantes J., Marote A., Ferreira S., Vieira S., Ferreira J. (2017). Biocompatibility and antimicrobial activity of biphasic calcium phosphate powders doped with metal ions for regenerative medicine. Ceram. Int..

[47] Wang K., Zhou C., Hong Y., Zhang X. (2012). A review of protein adsorption on bioceramics. Interface Focus.

[48] Cheng L., Ye F., Yang R., Lu X., Shi Y., Li L., Fan H., Bu H. (2010). Osteoinduction of hydroxyapatite/beta-tricalcium phosphate bioceramics in mice with a fractured fibula. Acta Biomater..

[49] Ebrahimi M., Botelho M.G., Dorozhkin S.V. (2017). Biphasic calcium phosphates bioceramics (HA/TCP): Concept, physicochemical properties and the impact of standardization of study protocols in biomaterials research. Mater. Sci. Eng. C.

[50] De Oliveira R.S., Brigato R., Madureira J.F.G., Cruz A.A.V., Filho F.V.D.M., Alonso N., Machado H.R. (2007). Reconstruction of a large complex skull defect in a child: A case report and literature review. Child’s Nerv. Syst..

[51] Bellucci D., Sola A., Gazzarri M., Chiellini F., Cannillo V. (2012). A new hydroxyapatite-based biocomposite for bone replacement. Mater. Sci. Eng. C.

[52] Popescu-Pelin G., Ristoscu C., Duta L., Stan G.E., Pasuk I., Tite T., Stan M.S., Bleotu C., Popa M., Chifiriuc M.C. (2020). Antimicrobial and Cytocompatible Bovine Hydroxyapatite-Alumina-Zeolite Composite Coatings Synthesized by Pulsed Laser Deposition from Low-Cost Sustainable Natural Resources. ACS Sustain. Chem. Eng..

[53] Roda M.A.P., Gilman E., Huntington T., Kennelly S.J., Suuronen P., Chaloupka M., Medley P.A. (2019). A Third Assessment of Global Marine Fisheries Discards. FAO Fisheries and Aquaculture Technical Paper.

[54] FAO (2018). The State of World Fisheries and Aquaculture 2018-Meeting the Sustainable Development Goals.

[55] González A.F., Gracia J., Miniño I., Romón J., Larsson C., Maroto J., Requeira M., Pascual S. (2018). Approach to reduce the zoonotic parasite load in fish stocks: When science meets technology. Fish. Res..

[56] Rustad T. (2003). Utilisation of marine by-products. Electron. J. Environ. Agric. Food Chem..

[57] Silva T., Moreira-Silva J., Marques A., Domingues A., Bayon Y., Reis R., Silva T.H., Moreira-Silva J., Marques A.L.P., Domingues A. (2014). Marine origin collagens and its potential applications. Mar. Drugs.

[58] Hamed I., Özogul F., Regenstein J.M. (2016). Industrial applications of crustacean by-products (chitin, chitosan, and chitooligosaccharides): A review. Trends Food Sci. Technol..

[59] Alves M.H.M.E., Nascimento G.A., Cabrera M.P., Silvério S.I., Da C., Nobre C., Teixeira J.A., de Carvalho L.B. (2017). Trypsin purification using magnetic particles of azocasein-iron composite. Food Chem..

[60] Lassoued I., Mora L., Nasri R., Jridi M., Toldrá F., Aristoy M.-C., Barkia A., Nasri M. (2015). Characterization and comparative assessment of antioxidant and ACE inhibitory activities of thornback ray gelatin hydrolysates. J. Funct. Foods.

[61] Maccari F., Galeotti F., Volpi N. (2015). Isolation and structural characterization of chondroitin sulfate from bony fishes. Carbohydr. Polym..

[62] Ciriminna R., Meneguzzo F., Delisi R., Pagliaro M. (2017). Enhancing and improving the extraction of omega-3 from fish oil. Sustain. Chem. Pharm..

[63] Ferraro V., Carvalho A.P., Piccirillo C., Santos M.M., Castro P.M., Pintado M.E. (2013). Extraction of high added value biological compounds from sardine, sardine-type fish and mackerel canning residues—A review. Mater. Sci. Eng. C.

[64] Toppe J., Albrektsen S., Hope B., Aksnes A. (2007). Chemical composition, mineral content and amino acid and lipid profiles in bones from various fish species. Comp. Biochem. Physiol. Part B Biochem. Mol. Biol..

[65] Chalamaiah M., Dinesh kumar B., Hemalatha R., Jyothirmayi T. (2012). Fish protein hydrolysates: Proximate composition, amino acid composition, antioxidant activities and applications: A review. Food Chem..

[66] Zamora-Sillero J., Gharsallaoui A., Prentice C. (2018). Peptides from fish by-product protein hydrolysates and its functional properties: An overview. Mar. Biotechnol..

[67] Ishak N.H., Sarbon N.M. (2018). A review of protein hydrolysates and bioactive peptides deriving from wastes generated by fish processing. Food Bioprocess Technol..

[68] Rustad T., Storrø I., Slizyte R. (2011). Possibilities for the utilisation of ma-rine by-products: Utilisation of marine by-products. Int. J. Food Sci. Technol..

[69] Adeoti I.A., Hawboldt K. (2014). A review of lipid extraction from fish processing by-product for use as a biofuel. Biomass Bioenergy.

[70] Olsen R.L., Toppe J., Karunasagar I. (2014). Challenges and realistic opportunities in the use of by-products from processing of fish and shellfish. Trends Food Sci. Technol..

[71] Moriguchi T., Nakagawa S., Kaji F. (2008). Reaction of Ca-deficient Hydroxyapatite with heavy metal ions along with metal substitution. Phosphorus Res. Bull..

[72] (1999). Annual Book of ASTM Standards, F 1581–1599. Standard Specification for Composition of Anorganic Bone for Surgical Implants.

[73] Griffith M., Islam M.M., Edin J., Papapavlou G., Buznyk O., Patra H.K. (2016). The Quest for Anti-inflammatory and Anti-infective Biomaterials in Clinical Translation. Front. Bioeng. Biotechnol..

[74] Rai M., Ingle A.P., Gaikwad S., Gupta I., Gade A., Silva S.S. (2016). Nanotechnology based anti-infectives to fight microbial intrusions. J. Appl. Microbiol..

[75] Létisse M., Comeau L. (2008). Enrichment of eicosapentaenoic acid and docosahexaenoic acid from sardine by-products by supercritical fluid fractionation. J. Sep. Sci..

[76] Murado M.A., Montemayor M.I., Cabo M.L., Vázquez J.A., González M.P. (2012). Optimization of extraction and purification process of hyaluronic acid from fish eyeball. Food Bioprod. Process..

[77] O’Sullivan A., Shaw N.B., Murphy S.C., van de Vis J.W., van Pelt-Heerschap H., Kerry J.P. (2006). Extraction of collagen from fish skins and its use in the manufacture of biopolymer films. J. Aquat. Food Prod. Technol..

[78] Sousa R.O., Martins E., Carvalho D.N., Alves A.L., Oliveira C., Duarte A.R.C., Silva T.H., Reis R.L. (2020). Collagen from Atlantic cod (Gadus morhua) skins extracted using CO_2_ acidified water with potential application in healthcare. J. Polym. Res..

[79] Sousa R.O., Alves A.L., Carvalho D.N., Martins E., Oliveira C., Duarte A.R.C., Silva T.H., Reis R.L. (2020). Acid and enzymatic extraction of collagen from Atlantic cod (Gadus Morhua) swim bladders envisaging health-related applications. Biomater. Sci. Polym..

[80] Seixas M.J., Martins E., Oliveira C., Duarte A.R.C., Silva T.H., Reis R.L. (2020). Extraction and characterization of collagen from elasmobranch byproducts for potential biomaterial use. Mar. Drugs.

[81] Herpandi H., Huda N., Adzitey F. (2011). Fish bone and scale as a potential source of halal gelatin. J. Fish. Aquat. Sci..

[82] Lv L.C., Huang Q.Y., Ding W., Xiao X.H., Zhang H.Y., Xiong L.X. (2019). Fish gelatin: The novel potential applications. J. Funct. Foods.

[83] Rafael M.Y.B., Rafael R.R., Landingin E.P., Rafael R.B., Tayag G.G., Santos J.P.E., Rafael M.J.R. (2021). Gelatin from Milkfish Scales for Food Application. CLSU Int. J. Sci. Technol..

[84] Venkatesan J., Qian Z.J., Ryu B., Thomas N.V., Kim S.K. (2011). A comparative study of thermal calcination and an alkaline hydrolysis method in the isolation of hydroxyapatite from Thunnus obesus bone. Biomed. Mater..

[85] Piccirillo C., Silva M., Pullar R., da Cruz I.B., Jorge R., Pintado M., Castro P.M. (2013). Extraction and characterisation of apatite-and tricalcium phosphate-based materials from cod fish bones. Mater. Sci. Eng. C.

[86] Goto T., Sasaki K. (2014). Effects of trace elements in fish bones on crystal characteristics of hydroxyapatite obtained by calcination. Ceram. Int..

[87] Piccirillo C., Pullar R.C., Tobaldi D.M., Castro P.M.L., Pintado M.M.E. (2014). Hydroxyapatite and chloroapatite derived from sardine by-products. Ceram. Int..

[88] Sunil B.R., Jagannatham M. (2016). Producing hydroxyapatite from fish bones by heat treatment. Mater. Lett..

[89] Pal A., Paul S., Choudhury A.R., Balla V.K., Das M., Sinha A. (2017). Synthesis of hydroxyapatite from Lates calcarifer fish bone for biomedical applications. Mater. Lett..

[90] Boutinguiza L.M., Pou Saracho J.M., Comesana Pineiro R., Lusquinos Rodriguez F., Leon Fong B.M. (2009). Biphasic Calcium Phosphate and Method for Obtaining Same from Fish Bones. European Patent Application.

[91] Kongsri S., Janpradit K., Buapa K., Techawongstien S., Chanthai S. (2013). Nanocrystalline hydroxyapatite from fish scale waste: Preparation, characterization and application for selenium adsorption in aqueous solution. Chem. Eng. J..

[92] Montañez Supelano N.D., Estupiñan H.A., Garcia S.J., Peña Ballesteros D.Y. (2018). Fabrication and characterization of novel biphasic calcium phosphate and nanosized hydroxyapatite derived from fish otoliths in different composition ratios. Chem. Eng. Trans..

[93] Zhu Q., Ablikim Z., Chen T., Cai Q., Xia J., Jiang D., Wang S. (2017). The Preparation and Characterization of HA/β-TCP Biphasic Ceramics from Fish Bones. Ceram. Int..

[94] Dou W., Chen H., Chen T., Zhu Q., Jiang D., Xue Z., Wang S., Wang S., Tang W. (2020). Design and construction of a microporous CO_3_^2−^-containing HA/β- TCP biphasic ceramic as a novel bone graft material. Mater. Res. Express.

[95] Kiyochi Junior H.d.J., Candido A.G., Bonadio T.G.M., Adauto da Cruz J., Baesso M.L., Weinand W.R., Hernandes L. (2020). In vivo evaluation of interactions between biphasic calcium phosphate (BCP)-niobium pentoxide (Nb_2_O_5_) nanocomposite and tissues using a rat critical-size calvarial defect model. J. Mater. Sci. Mater. Med..

[96] Piccirillo C., Dunnill C.W., Pullar R.C., Tobaldi D.M., Labrincha J.A., Parkin I.P., Pintado M.M., Castro P.M.L. (2013). Calcium phosphate-based materials of natural origin showing photocatalytic activity. J. Mater. Chem. A.

[97] Soumia B., Ahmed B., Abdelmjid A. (2017). Biphasic Calcium Phosphate Derived from a Sardine By-Product. Curr. Trends Biomed. Eng. Biosci..

[98] Bas M., Daglilar S., Kuskonmaz N., Kalkandelen C., Erdemir G., Kuruca S.E., Tulyaganov D., Yoshioka T., Gunduz O., Ficai D. (2020). Mechanical and Biocompatibility Properties of Calcium Phosphate Bioceramics Derived from Salmon Fish Bone Wastes. Int. J. Mol. Sci..

[99] Santosh Kumar B.Y., Isloor A.M., Anil S., Venkatesan J., Kumar G.C.M. (2019). Calcium phosphate bioceramics with polyvinyl alcohol hydrogels for biomedical applications. Mater. Res. Express.

[100] Piccirillo C., Pullar R.C., Costa E., Santos-Silva A., Pintado M.M.E., Castro P.M.L. (2015). Hydroxyapatite-based materials of marine origin: A bioactivity and sintering study. Mater. Sci. Eng. C.

[101] Basu S., Basu B. (2019). Unravelling Doped Biphasic Calcium Phosphate: Synthesis to Application. ACS Appl. Bio. Mater..

[102] Venkatesan J., Kim S.K. (2010). Effect of temperature on isolation and characterization of hydroxyapatite from tuna (Thunnus obesus) Bone. Materials.

[103] Piccirillo C., Adamiano A., Tobaldi D.M., Montalti M., Manzi J., Castro P.M.L., Panseri S., Montesi M., Sprio S., Tampieri A. (2017). Luminescent calcium phosphate bioceramics doped with europium derived from fish industry byproducts. J. Am. Ceram. Soc..

[104] Coelho T.M., Nogueira E.S., Steimacher A., Medina A.N., Weinand W.R., Lima W.M., Baesso M.L., Bento A.C. (2006). Characterization of natural nanostructured hydroxyapatite obtained from the bones of Brazilian river fish. J. Appl. Phys..

[105] Silva L.M., Rosso J.M., Bonadio T.G.M., Silva D.M., Dias G.S., Weinand W.R., Tominaga T.T., Miyahara R.Y., Cótica L.F., Santos I.A. (2018). On mechanical properties and bioactivity of PVDF-BCP composites. Cerâmica.

[106] Neto A.S., Fonseca A.C., Abrantes J.C.C., Coelho J.F.J., Ferreira J.M.F. (2019). Surface functionalization of cuttlefish bone-derived biphasic calcium phosphate scaffolds with polymeric coatings. Mater. Sci. Eng. C Mater. Biol. Appl..

[107] Pon-On W., Suntornsaratoon P., Charoenphandhu N., Thongbunchoo J., Krishnamra N., Tang I.M. (2016). Hydroxyapatite from fish scale for potential use as bone scaffold or regenerative material. Mater. Sci. Eng. C.

[108] Huang Y.-C., Hsiao P.-C., Chai H.-J. (2011). Hydroxyapatite extracted from fish scale: Effects on MG63 osteoblast-like cells. Ceram. Int..

[109] Fernández-Arias M., Álvarez-Olcina I., Malvido-Fresnillo P., Vázquez J.A., Boutinguiza M., Comesaña R., Pou J. (2021). Biogenic Calcium Phosphate from Fish Discards and By-Products. Appl. Sci..

[110] Bonadio T.G.M., Freitas V.F., Tominaga T.T., Miyahara R.Y., Rosso J.M., Cótica L.F., Baesso M.L., Weinand W.R., Santos I.A., Guo R. (2017). Polyvinylidene fluoride/hydroxyapatite/β-tricalcium phosphate multifunctional biocomposite: Potentialities for bone tissue engineering. Curr. Appl. Phys..

[111] Velten D., Eisenbarth E., Schanne N., Breme J. (2004). Biocompatible Nb_2_O_5_ thin films prepared by means of the sol-gel process. J. Mater. Sci. Mater. Med..

[112] Ribeiro C., Moreira S., Correia V., Sencadas V., Rocha J.G., Gama F.M., Ribelles J.L.G., Mendez S.L. (2012). Enhanced proliferation of pre-osteoblastic cells by dynamic piezoelectric stimulation. RSC Adv..

[113] Popescu-Pelin G., Ristoscu C., Duta L., Pasuk I., Stan G.E., Stan M.S., Popa M., Chifiriuc M.C., Hapenciuc C., Oktar F.N. (2020). Fish Bone Derived Bi-Phasic Calcium Phosphate Coatings Fabricated by Pulsed Laser Deposition for Biomedical Applications. Mar. Drugs.

[114] Behera R.R., Das A., Hasan A., Pamu D., Pandey L.M., Sankar M.R. (2020). Deposition of biphasic calcium phosphate film on laser surface textured Ti-6Al-4V and its effect on different biological properties for orthopedic applications. J. Alloy Compd..

[115] Chrisey D.B., Hubler G.K. (1994). Pulsed Laser Deposition of Thin Films.

[116] Eason R. (2006). Pulsed Laser Deposition of Thin Films-Applications-Led Growth of Functional Materials.

[117] Bao Q., Chen C., Wang D., Ji Q., Leia T. (2005). Pulsed laser deposition and its current research status in preparing hydroxyapatite thin films. Appl. Surf. Sci..

[118] Badea M., Braic M., Kiss A., Moga M., Pozna E., Pana I., Vladescu A. (2016). Influence of Ag content on the antibacterial properties of SiC doped hydroxyapatite coatings. Ceram. Int..

[119] Neto A.S., Brazete D., Ferreira J.M.F. (2019). Cuttlefish Bone-Derived Biphasic Calcium Phosphate Scaffolds Coated with Sol-Gel Derived Bioactive Glass. Materials.

[120] Venkatesan J., Rekha P., Anil S., Bhatnagar I., Sudha P., Dechsakulwatana C., Kim S.-K., Shim M.S. (2018). Hydroxyapatite from cuttlefish bone: Isolation, characterizations, and applications. Biotechnol. Bioprocess Eng..

[121] Ideia P., Esposti L.D., Miguel C.C., Adamiano A., Iafisco M., Castilho P.C. (2021). Extraction and characterization of hydroxyapatite-based materials from grey triggerfish skin and black scabbardfish bones. Int. J. Appl. Ceram. Technol..

[122] Montañez-Supelano N.D., Sandoval-Amador A., Estupiñan-Durán H.A., Peña-Ballesteros D.Y. (2017). A novel biphasic calcium phosphate derived from fish otoliths. J. Phys. Conf. Ser..

[123] Silva C.C., Pinheiro A.G., Figueiró S.D., Góes J.C., Sasaki J.M., Miranda M.A.R., Sombra A.S.B. (2002). Piezoelectric properties of collagen-nanocrystalline hydroxyapatite composites. J. Mater. Sci..

[124] Pollick S., Shors E.C., Holmes R.E., Kraut R.A. (1995). Bone formation and implant degradation of coralline porous ceramics placed in bone and ectopic sites. J. Oral Maxillofac. Surg..

[125] Ciobanu G., Ilisei S., Luca C., Carja G., Ciobanu O. (2012). The effect of vitamins to hydroxyapatite growth on porous polyurethane substrate. Prog. Org. Coat..

[126] Takeuchi A., Ohtsuki C., Miyazaki T., Kamitakahara M., Ogata S.-I., Yamazaki M., Furutani Y., Kinoshita H., Tanihara M. (2005). Heterogeneous nucleation of hydroxyapatite on protein: Structural effect of silk sericin. J. R. Soc. Interface.

[127] Yamada S., Heymann D., Bouler J.M., Daculsi G. (1997). Osteoclastic resorption of calcium phosphate ceramics with different hydroxyapatite/β-tricalcium phosphate ratios. Biomaterials.

[128] Cooper L.F., Zhou Y., Takebe J., Guo J., Abron A., Holmén A., Ellingsen J.E. (2006). Fluoride modification effects on osteoblast behavior and bone formation at TiO_2_ grit-blasted c.p. titanium endosseous implants. Biomaterials.

[129] Pazarçeviren A.E., Tezcaner A., Keskin D., Kolukısa S.T., Sürdem S., Evis Z. (2021). Boron-doped Biphasic Hydroxyapatite/β-Tricalcium Phosphate for Bone Tissue Engineering. Biol. Trace Elem. Res..

[130] Ebrahimi M., Pripatnanont P., Monmaturapoj N., Suttapreyasri S. (2012). Fabrication and characterization of novel nano hydroxyapatite/β-tricalcium phosphate scaffolds in three different composition ratios. J. Biomed. Mater. Res. A.

[131] LeGeros R.Z., Lin S., Rohanizadeh R., Mijares D., LeGeros J.P. (2003). Biphasic calcium phosphate bioceramics: Preparation, properties and applications. J. Mater. Sci. Mater. Med..

[132] Stock S.R. (2015). The Mineral–Collagen Interface in Bone. Calcif. Tissue Int..

[133] Habelitz S., Pascual L., Durán A. (2001). Transformation of tricalcium phosphate into apatite by ammonia treatment. J. Mater. Sci..

[134] Smičiklas I., Onjia A., Raičević S., Mitrić M. (2007). Factors influencing the removal of divalent cations by hydroxyapatite. J. Hazard. Mater..

[135] Gianmar D.E., Xiu L., Pasteris J.D. (2008). Immobilization of Lead with Nanocrystalline Carbonated Apatite Present in Fish Bone. Environ. Eng. Sci..

[136] Ozawa M., Suzuki M. (2002). Microstructural Development of Natural Hydroxyapatite Originated from Fish-Bone Waste through Heat Treatment. J. Am. Ceram. Soc..

[137] Lim J.J. (1975). Thermogravimetric analysis of human femur bone. J. Biol. Phys..

[138] Koutsopoulos S. (2002). Synthesis and characterization of hydroxyapatite crystals: A review study on the analytical methods. J. Biomed. Mater. Res..

[139] Yubao L., Wijn J.D., Klein C.P.A.T., Meer S.V., Groot K.D. (1994). Preparation and characterization of nanograde osteoapatite-like rod crystals. J. Mater. Sci. Mater. Med..

[140] Kannan S., Goetz-Neunhoeffer F., Neubauer J., Ferreira J.M.F. (2008). Ionic substitutions in biphasic hydroxyapatite and β-tricalcium phosphate mixtures: Structural analysis by Rietveld refinement. J. Am. Ceram. Soc..

[141] Dorozhkin S.V. (2013). Calcium orthophosphate-based bioceramics. Materials.

[142] Cesar R., Leivas T.P., Pereira C.A.M., Boffa R.S., Guarniero R., Reiff RBM Mandeli Netto A., Fortulan C.A., de Almeida Rollo J.M.D. (2017). Axial compressive strength of human vertebrae trabecular bones classified as normal, osteopenic and osteoporotic by quantitative ultrasonometry of calcaneus. Res. Biomed. Eng..

[143] Havaldar R., Pilli S.C., Putti B.B. (2014). Insights into the effects of tensile and compressive loadings on human femur bone. Adv. Biomed. Res..

[144] Karpiński R., Jaworski Ł., Czuback P. (2017). The structural and mechanical properties of the bone. J. Technol. Exploit Mech. Eng..

[145] Parente P., Savoini B., Ferrari B., Monge M.A., Pareja R., Sanchez-Herencia A.J. (2013). Effect of highly dispersed yttria addition on thermal stability of hydroxyapatite. Mater. Sci. Eng. C.

[146] Dorozhkin S.V. (2011). Biocomposites and hybrid biomaterials based on calcium orthophosphates. Biomatter.

[147] Canillas M., De Lima G.G., Rodríguez M.A., Nugent M.J.D., Devine D.M. (2015). Bioactive composites fabricated by freezing–thawing method for bone regeneration applications. J. Polym. Sci. Part B.

[148] Killion A., Geever L.M., Devine D.M., Farrell H., Higginbotham C.L. (2014). Compressive strength and bioactivity properties of photopolymerizable hybrid composite hydrogels for bone tissue engineering. Int. J. Polym. Mater. Polym. Biomater..

[149] Kenny E.K., Gately N.M., Killion J.A., Devine D.M., Higginbotham C.L., Geever L.M. (2016). Melt extruded bioresorbable polymer composites for potential regenerative medicine applications. Polym. Plast. Technol. Eng..

[150] Demirkol N., Oktar F.N., Kayali E.S. (2012). Mechanical and microstructural properties of sheep hydroxyapatite (SHA)–niobium oxide composites. Acta Phys. Pol. A.

[151] Duta L., Oktar F., Stan G., Popescu-Pelin G., Serban N., Luculescu C., Mihailescu I. (2013). Novel doped hydroxyapatite thin films obtained by pulsed laser deposition. Appl. Surf. Sci..

[152] Nelea V., Jelinek M., Mihailescu I.N., Eason R. (2007). Biomaterials: New issues and breakthroughs for biomedical applications. Pulsed Laser Deposition of Thin Films: Applications-Lead Growth of Functional Materials.

[153] Curcio M., De Stefanis A., De Bonis A., Teghil R., Rau J.V. (2019). Pulsed laser deposited bioactive RKKP-Mn glass-ceramic coatings on titanium. Surf. Coat. Technol..

[154] Von Euw S., Wang Y., Laurent G., Drouet C., Babonneau F., Nassif N., Azaïs T. (2019). Bone mineral: New insights into its chemical composition. Sci. Rep..

[155] Sponer P., Strnadova M., Urban K. (2011). In vivo behavior of low-temperature calcium-deficient hydroxyapatite: Comparison with deproteinised bovine bone. Int. Orthop..

[156] Dulski M., Dudek K., Grelowski M., Kubacki J., Hertlein J., Wojtyniak M., Goryczka T. (2018). Impact of annealing on features of BCP coating on NiTi shape memory alloy: Preparation and physicochemical characterization. Appl. Surf. Sci..

[157] Xia L., Xie Y., Fang B., Wang X., Lin K. (2018). In situ modulation of crystallinity and nano-structures to enhance the stability and osseointegration of hydroxyapatite coatings on Ti-6Al-4V implants. Chem. Eng. J..

[158] Thian E.S., Huang J., Best S.M., Barber Z.H., Bonfield W. (2005). Magnetron cosputtered silicon-containing hydroxyapatite thin films —An in vitro study. Biomaterials.

[159] Surmeneva M., Vladescu A., Surmenev R., Pantilimon C., Braic M., Cotrut C. (2016). Study on a hydrophobic Ti-doped hydroxyapatite coating for corrosion protection of a titanium based alloy. RSC Adv..

[160] Bohner M., Lemaitre J. (2009). Can bioactivity be tested in vitro with SBF solution?. Biomaterials.

[161] Kokubo T., Takadama H. (2006). How useful is SBF in predicting in vivo bone bioactivity?. Biomaterials.

[162] Popescu A., Florian P., Stan G., Popescu-Pelin G., Zgura I., Enculescu M., Oktar F., Trusca R., Sima L.E., Roseanu A. (2018). Physical-chemical characterization and biological assessment of simple and lithium-doped biological-derived hydroxyapatite thin films for a new generation of metallic implants. Appl. Surf. Sci..

[163] Hasan A., Pattanayek S.K., Pandey L.M. (2018). Effect of functional groups of selfassembled monolayers on protein adsorption and initial cell adhesion. ACS Biomater. Sci. Eng..

[164] Predoi D., Iconaru S.L., Predoi D., Stan G.E., Buton N. (2019). Synthesis, Characterization, and Antimicrobial Activity of Magnesium-Doped Hydroxyapatite Suspensions. Nanomaterials.

[165] Sutha S., Kavitha K., Karunakaran G., Rajendran V. (2013). In-vitro bioactivity, biocorrosion and antibacterial activity of silicon integrated hydroxyapatite/chitosan composite coating on 316L stainless steel implants. Mater. Sci. Eng. C.

[166] Rai M., Ingle A.P., Paralikar P. (2016). Sulfur and sulfur nanoparticles as potential antimicrobials: From traditional medicine to nanomedicine. Expert Rev. Anti-Infect. Ther..

[167] Rocha J.H.G., Lemos A.F., Agathopoulos S., Valério P., Kannan S., Oktar F.N., Ferreira J.M.F. (2005). Scaffolds for bone restoration from cuttlefish. Bone.

[168] Cao W., Hench L.L. (1996). Bioactive Materials. Ceram. Int..

[169] Hench L.L., Splinter R.J., Allen W.C., Greenlee T.K. (1971). Bonding mechanisms at the interface of ceramic prosthetic materials. J. Biomed. Mater. Res..

[170] Lowe B., Venkatesan J., Anil S., Shim M.S., Kim S.-K. (2016). Preparation and characterization of chitosan-natural nano hydroxyapatitefucoidan nanocomposites for bone tissue engineering. Int. J. Biol. Macromol..

[171] Nie L., Suo J., Zou P., Feng S. (2012). Preparation and properties of biphasic calcium phosphate scaffolds multiply coated with HA/PLLA nanocomposites for bone tissue engineering applications. J. Nanomater..

[172] Ou K., Dong X., Qin C., Ji X., He J. (2017). Properties and toughening mechanisms of PVA/PAM double-network hydrogels prepared by freeze-thawing and anneal-swelling. Mater. Sci. Eng. C.

[173] Dorozhkin S. (2015). Calcium orthophosphate-containing biocomposites and hybrid biomaterials for biomedical applications. J. Funct. Biomater..

[174] Rogina A., Ressler A., Matić I., Ferrer G.G., Marijanović I., Ivanković M., Ivanković H. (2017). Cellular hydrogels based on pH-responsive chitosan-hydroxyapatite system. Carbohydr. Polym..

[175] Chen F., Wang Z.C., Lin C.J. (2002). Preparation and characterization of nano-sized hydroxyapatite particles and hydroxyapatite/chitosan nano-composite for use in biomedical materials. Mater. Lett..

[176] Damien E., Revell P.A. (2004). Coralline hydroxyapatite bone graft substitute: A review of experimental studies and biomedical applications. J. Appl. Biomater. Biomech..

[177] Dorozhkin S.V. (2010). Bioceramics of calcium orthophosphates. Biomaterials.

[178] Peraire C., Arias J.L., Bernal D., Pou J., Leon B., Arano A., Roth W. (2006). Biological stability and osteoconductivity in rabbit tibia of pulsed laser deposited hydroxylapatite coatings. J. Biomed. Mater. Res. A.

[179] Boutinguiza M., Lusquiños F., Riveiro A., Comesaña R., Pou J. (2009). Hydroxylapatite nanoparticles obtained by fiber laser-induced fracture. Appl. Surf. Sci..

[180] Da Silva E.A.B., Costa C.A.E., Vilar V.J.P., Botelho C.M.S., Larosi M.B., Saracho J.M.P., Boaventura R.A.R. (2012). Water remediation using calcium phosphate derived from marine residues. Water Air Soil Pollut..

[181] Nzihou A., Sharrock P. (2010). Role of Phosphate in the Remediation and Reuse of Heavy Metal Polluted Wastes and Sites. Waste Biomass Valoriz..

[182] Piccirillo C., Fernández-Arias M., Boutinguiza M., Tobaldi D.M., Del Val J., Pintado M.M., Pou J. (2019). Increased UV absorption properties of natural hydroxyapatite-based sunscreen through laser ablation in liquid. J. Amer. Ceram. Soc..

[183] Atef M., Mahdi Ojagh S. (2017). Health benefits and food applications of bioactive compounds from fish byproducts: A review. J. Funct. Foods.

[184] Halim N.R.A., Yusof H.M., Sarbon N.M. (2016). Functional and bioactive properties of fish protein hydolysates and peptides: A comprehensive review. Trends Food Sci. Technol..

[185] He S., Franco C., Zhang W. (2013). Functions, applications and production of protein hydrolysates from fish processing co-products (FPCP). Food Res. Int..

[186] Adamiano A., Fellet G., Vuerich M., Scarpin D., Carella F., Piccirillo C., Jeon J.-R., Pizzutti A., Marchiol L., Iafisco M. (2021). Calcium Phosphate Particles Coated with Humic Substances: A Potential Plant Biostimulant from Circular Economy. Molecules.

